# Bioinformatics-Driven Identification and Examination of Candidate Genes for Non-Alcoholic Fatty Liver Disease

**DOI:** 10.1371/journal.pone.0016542

**Published:** 2011-01-27

**Authors:** Karina Banasik, Johanne M. Justesen, Malene Hornbak, Nikolaj T. Krarup, Anette P. Gjesing, Camilla H. Sandholt, Thomas S. Jensen, Niels Grarup, Åsa Andersson, Torben Jørgensen, Daniel R. Witte, Annelli Sandbæk, Torsten Lauritzen, Bernard Thorens, Søren Brunak, Thorkild I. A. Sørensen, Oluf Pedersen, Torben Hansen

**Affiliations:** 1 Hagedorn Research Institute, Gentofte, Denmark; 2 Institute of Biomedical Sciences, University of Copenhagen, Copenhagen, Denmark; 3 Center for Biological Sequence Analysis, Technical University of Denmark, Lyngby, Denmark; 4 Faculty of Pharmaceutical Sciences, University of Copenhagen, Copenhagen, Denmark; 5 Research Centre for Prevention and Health, Glostrup University Hospital, Glostrup, Denmark; 6 Steno Diabetes Center, Gentofte, Denmark; 7 Department of General Practice, University of Aarhus, Aarhus, Denmark; 8 Institute of Pharmacology and Toxicology, University of Lausanne, Lausanne, Switzerland; 9 Institute of Preventive Medicine, Copenhagen, University Hospital, Center for Health and Society, Copenhagen, Denmark; 10 Faculty of Health Sciences, University of Aarhus, Aarhus, Denmark; 11 Faculty of Health Sciences, University of Southern Denmark, Odense, Denmark; University of Georgia, United States of America

## Abstract

**Objective:**

Candidate genes for non-alcoholic fatty liver disease (NAFLD) identified by a bioinformatics approach were examined for variant associations to quantitative traits of NAFLD-related phenotypes.

**Research Design and Methods:**

By integrating public database text mining, trans-organism protein-protein interaction transferal, and information on liver protein expression a protein-protein interaction network was constructed and from this a smaller isolated interactome was identified. Five genes from this interactome were selected for genetic analysis. Twenty-one tag single-nucleotide polymorphisms (SNPs) which captured all common variation in these genes were genotyped in 10,196 Danes, and analyzed for association with NAFLD-related quantitative traits, type 2 diabetes (T2D), central obesity, and WHO-defined metabolic syndrome (MetS).

**Results:**

273 genes were included in the protein-protein interaction analysis and *EHHADH, ECHS1, HADHA, HADHB*, and *ACADL* were selected for further examination. A total of 10 nominal statistical significant associations (*P*<0.05) to quantitative metabolic traits were identified. Also, the case-control study showed associations between variation in the five genes and T2D, central obesity, and MetS, respectively. Bonferroni adjustments for multiple testing negated all associations.

**Conclusions:**

Using a bioinformatics approach we identified five candidate genes for NAFLD. However, we failed to provide evidence of associations with major effects between SNPs in these five genes and NAFLD-related quantitative traits, T2D, central obesity, and MetS.

## Introduction

It has been estimated that around 20% of all adults have non-alcoholic fatty liver disease (NAFLD) [1], [2], which is defined by accumulation of fat in hepatocytes exceeding 5-10% of the liver weight [3]. In the obese adult population NAFLD is present among 60% [1], [2]. NAFLD associates with insulin resistance and type 2 diabetes (T2D) and it has been suggested that it might predict the presence or future development of the metabolic syndrome (MetS) [4], [5]. While environmental factors causing NAFLD are well-known [3], [6], it has been suggested that genetics factors also predispose to NAFLD [7], [8] and that these might explain the difference in NAFLD progression between individuals [7], [9].

At the biochemical level NAFLD often presents with abnormal liver enzymes without the presence of markers of other common liver disease, e.g., hepatitis C [10]. Non-invasive tests such as the BAAT (body mass index, age, alanine aminotransferase, triglycerides), the European liver fibrosis score, Fibrotest, Fibroscan, hyaluronic acid, BARD (body mass index, aspartate aminotransferase (AST):alanine aminotransferase (ALT), diabetes), non-alcoholic steatohepatitis (NASH) score, and the NAFLD fibrosis score have been developed [11], and have all been tested in individuals with NAFLD [10]. However, these tests are still insufficient to replace liver biopsy [10], which currently is the gold standard for the diagnosis and assessment of severity of NAFLD [3]. Nevertheless, knowledge of MetS, T2D, serum insulin, AST, and ALT concentrations has in a study by Kotronen *et al.* allowed prediction of NAFLD with a sensitivity of 86% and a specificity of 71% [12].

Several genes have been suggested as putative candidate genes for NAFLD susceptibility or progression of the disease [7]. Recently, genome-wide association (GWA) studies have successfully contributed to the gene discovery process by identifying common genetic variants in several complex human diseases including NAFLD. In a GWA study of liver fat content in 2,111 individuals of different ancestry, the G-allele of rs738409 in patatin-like phospholipase domain containing 3 (*PNPLA3*) showed strong evidence of association with NAFLD [13]. Additionally, *PNPLA3* was found to be associated with ALT concentration in a GWA of plasma liver-enzyme levels in a Caucasian population [14]. However, addition of information of variation in *PNPLA3* did not significantly improve the NAFLD prediction score [12].

Bioinformatics is often used in the investigation, establishment, and ranking of biological candidate genes, and e.g., protein-protein interaction analyses can be used to identify disease-related complexes [15]. This method places the potential disease-causing proteins in a functional context, relative to other known or unknown disease-associated proteins, and thus, systematic investigation of such complexes might unmask new candidate genes for NAFLD-related phenotypes.

The objective of the present study was to identify new putative candidate genes for NAFLD-associated phenotypes using a bioinformatics approach that implements text mining, trans-organism protein-protein interaction transferal, and publicly available information on protein expression levels in the liver. Furthermore, we investigated the association of common genetic variants in the candidate genes with NAFLD-related quantitative traits (waist circumference, serum triglyceride, and fasting levels of serum insulin and plasma glucose) in 6,162 middle-aged Danes. Also, case-control analyses were performed using a total of 10,196 middle-aged Danes to investigate putative associations between the genetic variants and T2D, central obesity, and MetS.

## Materials and Methods

The studies were approved by the Ethical Committee of Copenhagen and were in accordance with the principles of the Declaration of Helsinki II. Informed written consent was obtained from all individuals before participation.

### Selection of candidate genes using a bioinformatics approach

We used text mining in NCBI Databases (build 36) as the initial approach to identify potential biological candidate genes. This consisted of two PubMed searches using the search terms “(“hepatic steatosis” OR “NAFLD”) AND genes”, or “(“visceral obesity” OR “waist circumference”) AND genes”. Limits: only within year 2003–2008 that reduces the search results with app. 50%. All results were reviewed manually. Only papers published in English were considered. Full texts of papers were searched when accessible. Also, we searched the online mendelian inheritance in man (OMIM) for genetic abnormalities leading to syndromes with fatty liver related phenotypes, and for the terms “(“hepatic steatosis” OR “NAFLD”) AND genes”, or “(“visceral obesity” OR “waist circumference”) AND genes”. Text mining identified 273 genes putatively implicated in NAFLD, and these were prioritized according to how often they were co-mentioned with the terms “fatty liver” or “NAFLD” in the literature. Of the 273 genes we removed 26 genes due to gene symbol overlap, missing human homologue or missing ensembl ID. The 247 remaining biological candidate genes were then prioritized using a scoring scheme based on the origin of the data and OMIM phenotypes (Supporting Information S1). 37 biological candidate genes had a priority score >2 (Supporting Information S1) and were selected for further bioinformatics analysis according to: 1) MeSH terms (using the GeneCard database (www.genecards.org)), 2) keywords (using the AKS2 database (www.bioalma.com/aks2/index.php)), 3) pathway analysis (investigating e.g., biological processes, functional role and sub cellular localization using the KEGG database (www.genome.jp/kegg/)), 4) interactomes (a protein-protein interaction analysis with trans-organism protein-protein interaction transferal) [15], and 5) the GNF expression profiles from healthy tissues [16], [17].

**Figure 1:**
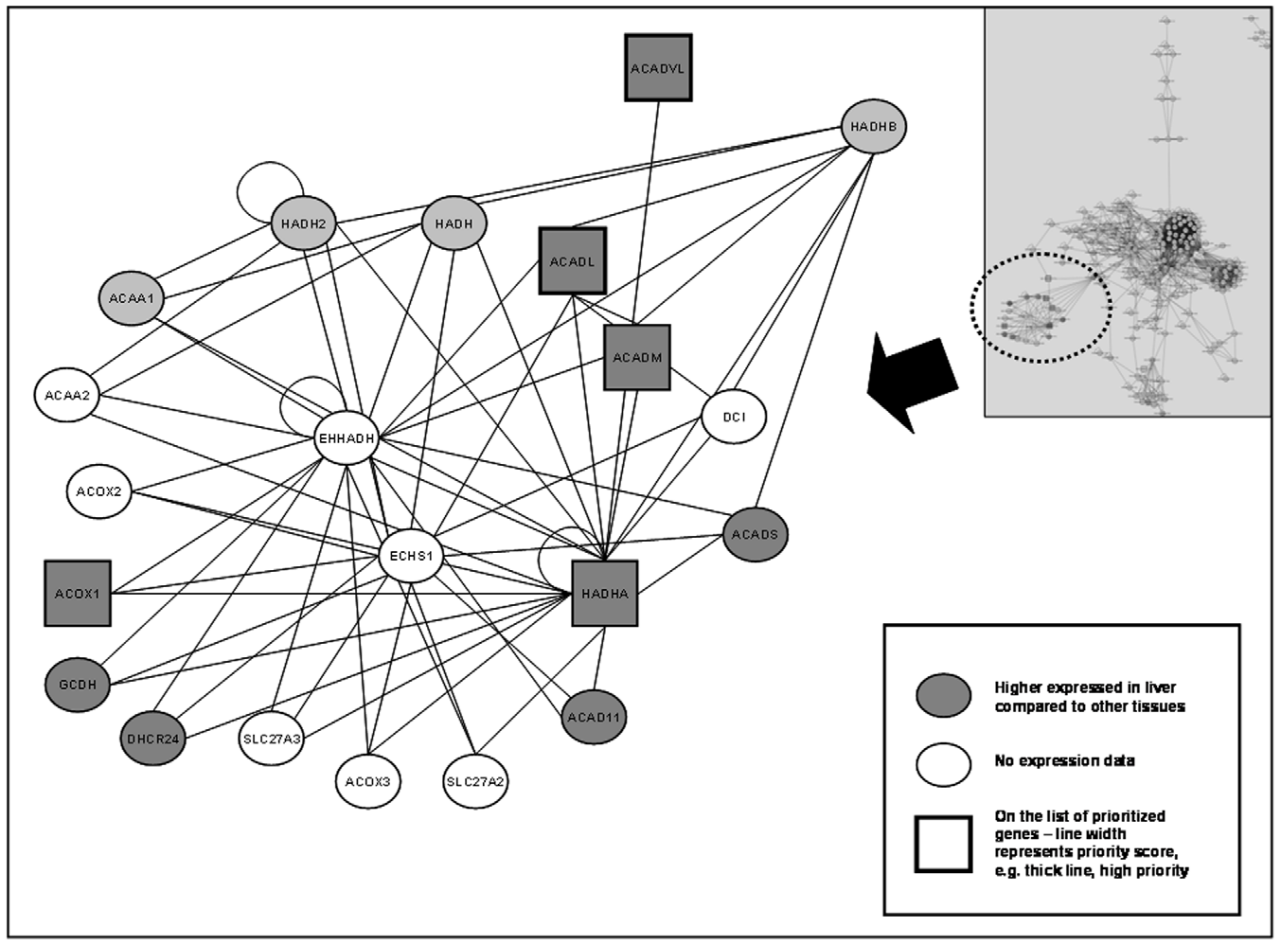
Interactome from the protein-protein interaction analysis. Enlarged picture of the interactome, from which *EHHADH, ECHS1, HADHA, HADHB*, and *ACADL* were selected for further analyses. Nodes are round if not on the list of prioritized candidate genes, and squared if on the list. The thickness of the line represents the priority score. The thicker the line, the higher prioritized on the list of candidate genes. Dark background color means highly expressed (above average) in liver compared to other tissues in the renormalized tissue expression data set [17]. White nodes represent proteins with no expression data available in the renormalized data set. However, in the orginal GNF tissue data set [16], all the genes corresponding to the white proteins have an expression level above the median for all tissues, supporting the observation that the proteins have a relative higher expression in liver. In the upper right corner is the entire protein-protein interaction network depicted. The smaller interactome is highlighted with a dashed circle. Cytoscape 2.6 (http://www.cytoscape.org/) was used to visualize the interactome.

One interactome contained 5 of our 37 biological candidate genes (Figure 1). Five genes – peroxisomal bifunctional enzyme (*EHHADH*), enoyl-CoA hydratase, mitochondrial (*ECHS1*), long-chain specific acyl-CoA dehydrogenase, mitochondrial (*ACADL*), trifunctional enzyme subunit alpha, mitochondrial (*HADHA*), and trifunctional enzyme subunit beta, mitochondrial (*HADHB*) – were chosen from this interactome for further investigation, based on the following criteria: 1) centrally placed in the interactome; 2) five or more connections to other nodes in the network; and, if available, evidence of high levels of expression in the liver compared to other tissues.

### Selection of SNPs for genotyping

According to HapMap phase II (release 24), 21 tagSNPs (CEU) capture all variation in these genes (±10 kb) at an *r* ^2^ threshold of at least 0.8 (MAF between 1 and 45%) and were chosen for genotyping. Since *HADHA* and *HADHB* share chromosomal location and therefore are tagged by the same variants, they will from now on be referred to as *HADHA*/*B* and analyzed together. Haploview (version 4.2) was used to construct linkage disequilibrium (LD) plots displaying r^2^ between each genotyped variant in a locus (Supporting Information S1).

### Genotyping

The tagSNPs were genotyped using KASPar® (KBiosciences, UK) with success rates >96% and error rates not exceeding 0.5% (>1,177 replicates). Genotype distributions obeyed Hardy-Weinberg equilibrium (*P*>0.05) in all study groups, except for rs1056471 (*P* = 0.05) and rs3791731 (*P* = 0.007) in *HADHA/B*; and rs6805633 (*P* = 4×10^−27^) in *EHHADH*. These three SNPs were excluded from the analyses.

### Study participants

This study involved 10,196 unrelated Danes from four study groups. Details of the study samples are given in Supporting Information S1. 1) The population-based sample (Inter99) of middle-aged individuals (*n* = 6,162) sampled at the Research Centre for Prevention and Health [18]. 2) T2D patients sampled through the out-patient clinic at Steno Diabetes Center (SDC) (*n* = 1,695). 3) A population-based group of middle-aged glucose-tolerant participants recruited via SDC (*n* = 730). Finally, the ADDITION Denmark study (Anglo-Danish-Dutch Study of Intensive Treatment in People with Screen-Detected Diabetes in Primary Care) (ClinicalTrials.gov ID-no: NCT00237548 [19], which is a population-based, high-risk screening and intervention study for T2D in general practice (*n* = 1,609) was included as the fourth group. All participants in study group 1 and 3 underwent a standard 75 g oral glucose tolerance test (OGTT). T2D and glucose tolerance was diagnosed according to the World Health Organization (WHO) 1999 criteria [20], and central obesity was defined using waist-circumference (≥88 cm for women and ≥102 cm for men).

Quantitative trait analyses were carried out in glucose tolerant individuals from study group 1 (*n = *4,567), whereas the case-control studies of T2D and central obesity involved all four study groups (*n* = 10,196). Individuals with MetS were identified from study group 1. The case-control study of MetS was defined according to the 1998 WHO criteria [21] and involved 1,757 control individuals and 1,349 individuals with either impaired glucose tolerance (defined as increased fasting glycaemia (IFG), impaired glucose tolerance (IGT), screen-detected T2D (scT2D), or T2D), or increased insulin resistance calculated using the homeostasis model assessment of insulin resistance (HOMA-IR), together with two or more components of MetS (dyslipidemia, hypertension, obesity, or albuminuria). Control individuals were defined as not having any of the components comprised in the WHO-defined criteria of MetS.

### Selection of NAFLD-related phenotypes

NAFLD-related quantitative traits that were investigated in this study included: waist circumference, serum triglyceride, and fasting levels of serum insulin and plasma glucose. These traits were considered the best surrogate measures of NAFLD based on previous studies [12], [22].

### Anthropometrical and biochemical measurements

Height (without shoes) and weight were measured in light indoor clothing, and BMI was calculated as weight in kg/(height in m)^2^. Waist circumference was measured in the upright position midway between the iliac crest and the lower costal margin [18]. Blood samples were drawn after a 12 h overnight fast. Plasma glucose was analyzed by a glucose oxidase method (Granutest; Merck, Darmstadt, Germany) and serum insulin (excluding des-31,32 and intact proinsulin) was measured using the Autodelfia insulin kit (Perkin-Elmer/Wallac, Turku, Finland). Serum triglycerides were analyzed using enzymatic colorimetric methods (GPO-PAP and CHOD-PAP; Roche Molecular Biochemicals, Mannheim, Germany). HOMA-IR was calculated as: (fasting plasma glucose (mmol/l) × fasting serum insulin (pmol/l))/22.5 [23].

### Statistical analyses

Analyses were performed using R version 2.10.0. *P*-values were not adjusted for multiple hypothesis testing and *P*<0.05 was considered significant.

#### Association with NAFLD-related phenotypes

Quantitative trait studies were performed using a general linear model. Data with non-normally distributed residuals (serum triglycerides and serum insulin release) were logarithmically transformed prior to analyses. All analyses were adjusted for age, sex, and BMI, assuming an additive model (*P*_add_). Effect sizes are denoted as β that reflect a per allele effect with 95% confidence interval (CI), and are given as actual values or percentage if logarithmically transformed. The statistical power was estimated using 1,000 simulations and a significance threshold of 0.05. Based on the allele frequencies of the variant and the sample size of 6,162 individuals, we have estimated the effect sizes per allele of quantitative traits for which we had 60 and 90% statistical power, respectively, to detect an association. Power estimates for the quantitative trait analyses are summarized in Supporting Information S1. A quantile–quantile (QQ) plot was generated by plotting the observed ordered allele associations from the quantitative trait analyses against the ordered expected associations (Supporting Information S1).

To investigate higher order interactions between the 18 SNPs and environmental factors we applied the software package Bayesian Association for Multiple SNP Effects (BAMSE) [24], that model gene-environment interactions while accounting for multiple testing. We used the Inter99 study population including the 18 SNPs and glucose tolerance status (NGT, IFG, IGT, and scT2D), as well as questionnaire-based information on four self-reported environmental factors: physical activity (passive, light or medium, and hard or very hard), energy intake (kJ/day), alcohol intake (gram/day), and smoking habits (daily, occasionally, ex-smoker or never) to test if any of these associated with any of the investigated quantitative traits.

#### T2D, central obesity, and MetS association analyses

Logistic regression was used to examine differences in genotype distribution in the case-control studies. The T2D case-control analyses were adjusted for age, sex, and BMI. We included 2,330 T2D patients in the case-control for central obesity, why these analyses were adjusted for age, sex, BMI, and diabetes treatment. The MetS case-control analyses were adjusted for age and sex. The statistical power calculations in the case-control studies were done using CaTS, power calculations for large genetic association studies, available at http://www.sph.umich.edu/csg/abecasis/cats/. The statistical power to detect an OR of 1.10 was, depending on the investigated trait, estimated to be between 48 and 99% for variants with a MAF>20%. The statistical power for the case-control analyses is summarized in Supporting Information S1.

### Functional prediction for the variants

The potential functional effects of the variants were predicted using Ensembl SNP Effect Predictor (http://www.ensembl.org/) and FastSNP [25].

## Results

### Selection of candidate genes

Text mining identified 273 genes putatively implicated in NAFLD-related phenotypes, of which 37 biological candidate genes where selected for further analysis. From these genes *EHHADH, ECHS1, ACADL, HADHA*, and *HADHB*, which are involved in the mitochondrial fatty acid β-oxidation, were selected as candidate genes for tagging and genotyping (Figure 1).

### Studies of associations to NAFLD-related quantitative traits

**Figure 2:**
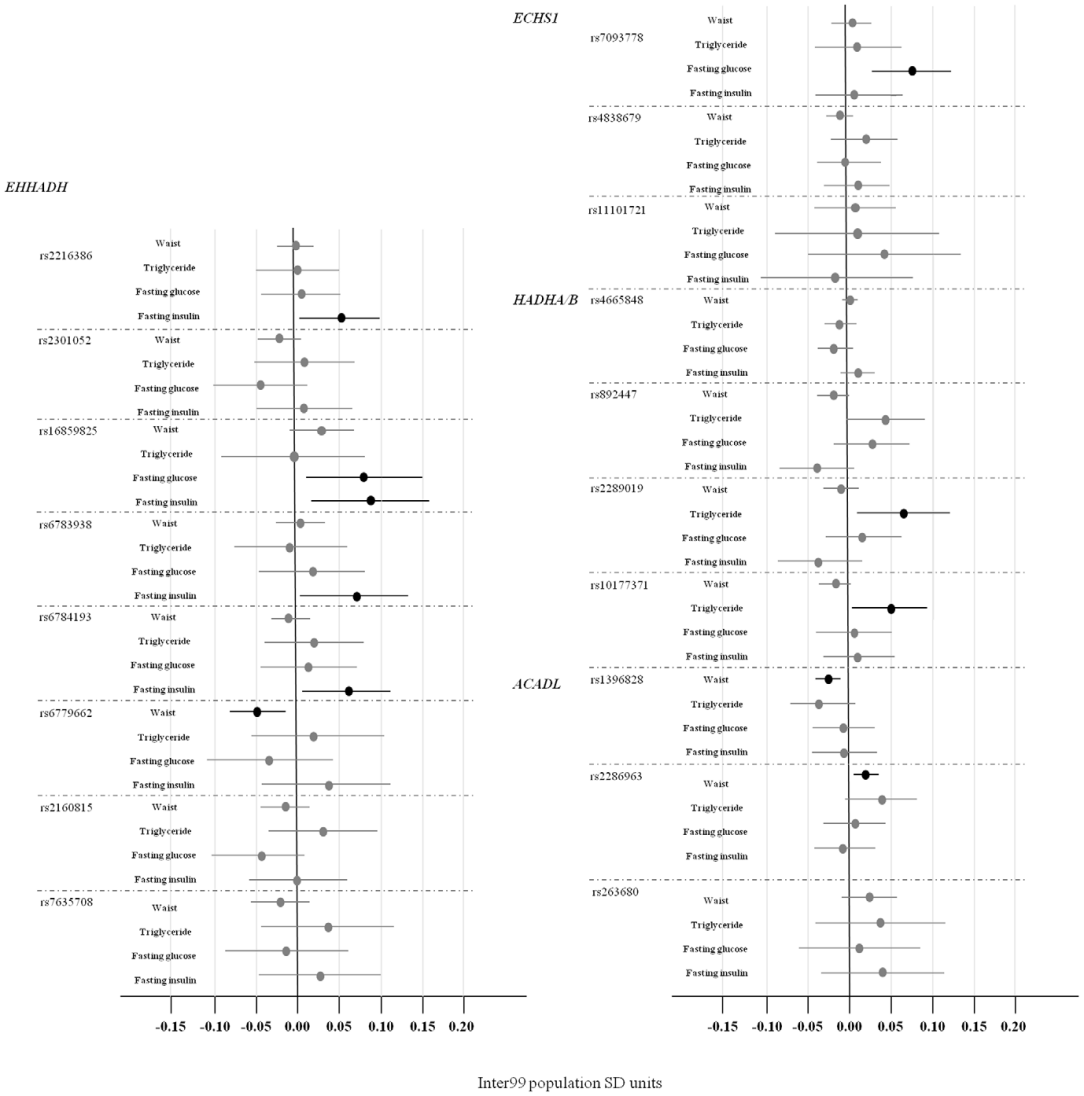
Quantitative trait analyses of NAFLD-related traits in (*n* = 4,567) glucose-tolerant Danes. Standardized Inter99 population SD units for NAFLD-related traits: waist circumference, fasting serum triglycerides, fasting plasma glucose, and fasting serum insulin. Calculated as mean(trait)/SD(trait). The analyses were adjusted for age, sex, and BMI.

Figure 2 summarizes the results for the four NAFLD–related quantitative traits: waist circumference, fasting serum triglyceride, fasting plasma glucose, and fasting serum insulin; values are given in comparable Inter99 population SD units. Nine genetic variants showed significant associations with one or more of these traits (Table 1). Extensive results from the quantitative association analysis are enclosed in Supporting Information S1. A QQ-plot to visualize the distribution of observed versus expected *P*-values from the quantitative trait analyses, is enclosed in Supporting Information S1.

**Table 1:** Nominal statistically significant associations with NAFLD-related traits in the quantitative trait analyses in (*n* = 4,567) glucose-tolerant Danes.

Gene	SNP	Major/minor allele	Trait	*n*WT/HE/HO	Per allele effect (95%CI)	*P*_additive_
*EHHADH*	rs2216386	A/G	Fasting serum insulin	2777/1406/180	3.4% (0.8%; 6.1%)	0.01
*EHHADH*	rs16859825	T/C	Fasting plasma glucose (mmol/l)	3747/613/24	0.033 (0.004;0.062)	0.03
			Fasting serum insulin		5.3% (1.4%;9.3%)	0.01
*EHHADH*	rs6783938	C/T	Fasting serum insulin	3596/765/39	4.1% (0.5%; 7.7%)	0.03
*EHHADH*	rs6784193	A/G	Fasting serum insulin	3089/1157/107	3.8% (0.8%; 6.7%)	0.01
*EHHADH*	rs6779662	T/C	Waist circumference (cm)	3786/542/21	−0.60 (−1.03; −0.17)	0.006
*EHHADH*	rs2160815	T/A	Serum triglycerides	3397/925/67	3.2% (0.3%; 6.1%)	0.03
*ECHS1*	rs7093778	T/C	Fasting plasma glucose (mmol/l)	840/1350/149	0.03 (0.01; 0.05)	0.002
*ACADL*	rs1396828	T/C	Waist circumference (cm)	1267/2139/974	−0.3 (−0.5; −0.1)	0.01
*ACADL*	rs2286963	T/G	Waist circumference (cm)	1825/1933/569	0.26 (0.04; 0.48)	0.02

Values of serum triglycerides and serum insulin were logarithmically transformed prior to statistical analyses, and their effect sizes are presented as the increase/decrease in percent. Effect sizes and *P*-values shown are for an additive genetic model and are adjusted for age, sex, and BMI. WT, wild-type. HE, heterozygous. HO, homozygous.

Investigating higher order interactions between the 18 SNPs and environmental factors using BAMSE showed no significant associations.

### T2D, central obesity, and MetS case-control studies

Two variants in *EHHADH* were associated with T2D: rs6784193 (OR_add_ = 1.14(1.01–1.29), *P*_add_ = 0.03) and rs7635708 (OR_add_ = 1.20(1.02–1.41), *P*_add_ = 0.03). We found no association with T2D for any of the variants in *ECHS1, ACADL,* or *HADHA/B* (Supporting Information S1).

The minor G-allele of rs11101721 in *ECHS1* associated with central obesity (OR_add_ = 1.21(1.05–1.40), *P*_add_ = 0.008). For *HADHA/B*, the minor G-allele associated with a decrease in risk of developing central obesity (OR_add_ = 0.93(0.87–0.99), *P*_add_ = 0.03). For the other variants, no statistically significant associations were observed when comparing the genotype distribution between lean individuals and obese individuals as defined by waist circumference (Supporting Information S1).

When comparing the genotype distribution between individuals with MetS and individuals with no clinical evidence of MetS, the minor G-allele of rs11101721 in *ECHS1* associated with MetS (OR_add_ = 1.20(1.01–1.43), *P*_add_ = 0.04). No variants in *EHHADH*, *ACADL*, or *HADHA/B* showed associations with MetS (Supporting Information S1).

## Discussion

We aimed at identifying new putative candidate genes for NAFLD-related phenotypes by applying trans-organism protein-protein interaction transferal combined with liver expression data, and to investigate genetic variants in the candidate genes and their association with metabolic traits known to relate to NAFLD.

The main findings of this study were: 1) the bioinformatics approach proved successful in identifying putative candidate genes, 2) the variations in the selected candidate genes were associated with several NAFLD-associated traits, suggesting a potential role in the development of these metabolic phenotypes.

### Selection of candidate genes

We identified five genes involved in the β-oxidation of fatty acids. The activities of mitochondrial respiratory chain enzyme complexes have been shown to be impaired in the liver of patients with NASH [26], suggesting that mitochondrial dysfunction might play a role in the pathogenesis of NAFLD. However, it has not yet been clarified which molecular mechanisms cause the defects. A common denominator of the five identified genes is peroxisome proliferator-activated receptor alpha (PPARα), which play a role in regulation of the genes. PPARα has previously been investigated in our study populations in relation to T2D- and obesity-related quantitative traits [27], but has also been suggested as a gene predisposing for NAFLD [28]. PPARα is the main regulator of fatty acid utilization [29] and when activated, PPARα causes a decrease in plasma-, hepatic-, and intramuscular contents of triglycerides [30]. *EHHADH* binds to PPARα providing a positive feed-back loop, thereby adjusting the tissue expression levels of PPARα according to a given metabolic need [31]. In rats, down-regulation of *ECHS1* has been identified as a contributing factor in high-fat diet induced hepatic steatosis, causing decreased mitochondrial fatty acid β-oxidation, a finding that was validated in patients with simple steatosis [32]. Furthermore, it was shown that down-regulation of *ECHS1* by small interfering RNA (*in vitro* and *in vivo*) aggravated the accumulation of lipids in hepatocytes caused by free fatty acid overload [32]. Moreover, PPARα responsive elements have been identified in *ECHS1* [33], but it remains to be determined whether the decreased mitochondrial fatty acid β-oxidation induced by *ECHS1* down-regulation is mediated through PPARα.

PPARα agonists used to treat dyslipidemia have been shown to moderately induce the expression of *ACADL* and *ECHS1* in rat liver [34], and in rat heart muscle PPARα agonists increase the expression of *ECHS1* and reduce the expression of *HADHA*/*B* [35]. These findings may indicate that PPARα also regulates these genes. Furthermore, treatment with a PPARα agonist improved steatosis in fatty liver Shionogi mice and reduced hepatic triglyceride level by inducing expression of several genes involved in the turnover of fatty acids, e.g., *ACADL* [36]. In a study of 26 histology diagnosed NAFLD patients, liver expression of both *ACADL* and *HADHA* was increased 3-6-fold compared to healthy individuals [28]. In contrast, Eaton et *al*. showed that patients with NAFLD had a significantly increased amount of 3-hydroxyacyl-CoA and 2-enoyl-CoA esters in their liver mitochondria, suggesting a decreased *HADHA* activity [37].

Investigating a potential functional role of the variants included in this study, revealed that rs2286936 of *ACADL* is a non-synonymous coding variant and thereby could impact on the measured phenotypes. This variant has been shown to associate with the metabolite acylcarnitine 9 in a GWA study of 163 metabolic traits measured in human blood (initial step: *n* = 1,809/replication step: *n* = 422), indicating that this variant may play a role in human lipid metabolism [38]. None of the other variants are coding variants, but thorough functional characterization is warranted to eliminate other mechanisms in which these variants might alter the phenotype. Taken into account the previous knowledge about the five selected genes, we propose that they are reasonable candidate genes, and therefore hypothesize that they may play a role in the development in NAFLD and NAFLD-related metabolic traits.

The NAFLD-associated *PNPLA3* was not directly identified using this approach, but the glycerolipid metabolism pathway that *PNPLA3* is involved in, is connected to the fatty acid metabolism pathway, which was one of the top ranked pathways in the pathway analysis.

### Association with NAFLD-associated quantitative traits

Variation in several of the genes associated with the investigated quantitative traits that relate to NAFLD, indicating that these genes, alone or in combination, might influence the development and/or progression of NAFLD. However, no evidence of associations of major effects between these variants and NAFLD-related quantitative traits was found. We constructed a QQ-plot to visualize the distribution of observed versus expected *P*-values from the quantitative trait analyses. Limits to this method are the low number of observations, potentially high LD between some of the variants, and non-normally distributed data. Despite this, the QQ-plot showed an over-abundance of low *P*-values (Supporting Information S1). Given the relatively high LD between some of the variants in a locus that shows associations with a trait, e.g., rs6783938 and rs6784193 of *EHHADH* with r^2^ = 0.7 which both are associated with fasting serum insulin, we cannot preclude that these associations reflect the same causative variant. None of the investigated variants were identified in the recent GWA studies of NAFLD or surrogate NAFLD measures such as plasma levels of liver enzymes [13], [14]. Hence, investigating the effect of these variants in combination with other genetic variants or environmental factors would help elucidating their potential role in NAFLD. However, examining higher order interactions between the variants and environmental factors in the present study applying the software package BAMSE did not show any combinations that significantly contributed to the investigated NAFLD-related phenotypes (data not shown).

### T2D, central obesity, and MetS case-control studies

Only few of the variants showed association to T2D, MetS, or central obesity. This is, however, not tantamount to them being irrelevant for the development of NAFLD. Here, we assume that individuals with T2D, MetS, or central obesity suffer from some degree of NAFLD, but we have no clinical evidence of liver disease in these patients. Therefore, an alternative study design would be to focus on search for associations of these variants with the mentioned metabolic disorders in cases with clinically diagnosed NAFLD.

The strengths of this study lie within the chosen methods. Text mining and the other bioinformatics approaches used take advantage of data available from public sources, thereby implementing many different types of studies. Many diseases that present a similar clinical phenotype are caused by variation in genes that are part of the same functional protein complex, where the overlapping phenotype can be explained by variation in single genes, or combinations of genes, making the entire protein complex dysfunctional [15]. Also, the large well-characterized study samples used, in most cases provide sufficient statistical power (>80%) to detect moderate genetic effects on quantitative surrogate measures of NAFLD (Supporting Information S1).

One could speculate that the text mining approach would discriminate in favor of the included genes, and that previous studies investigating the role of variation in these genes in relation to NAFLD, contribute to their status as highly prioritized candidate genes. This has been taken into consideration by the subsequent protein-protein interaction analysis, where interacting proteins were included to the list of prioritized candidates. From the interactome, only *ACADL* and *HADHA* were present on the prioritized list, and we therefore believe that discrimination has been minimized.

There are additional limitations to this study. Text mining was limited to PubMed and OMIM only and, additionally, to a limited time-period, meaning that we may have missed important information reported elsewhere or at a different time-point. Also, text mining was itself limited, as it did not include full-text articles. However, we believe that we have accounted for this by applying the other steps in the bioinformatics approach, in which we extracted additional unlimited information from several other sources.

Here we investigate the impact of potential candidate genes on surrogate measures of NAFLD. The use of surrogate measures is experimental and should be interpreted with caution. Furthermore, it should be noted that information on alcohol consumption within the study population is based on questionnaires, which introduces another bias to this study, and the possibility that excessive alcohol abuse was present in some of the investigated individuals cannot be eliminated. Finally, substantial multiple testing correction is needed to account for statistical type I errors. In the present study, Bonferroni adjustments for multiple testing negate all associations.

Using a bioinformatics approach we have identified five new potential candidate genes for NAFLD. Our exploratory analyses suggest that these genes may contribute to the development of NAFLD; however, we failed to provide evidence of associations with major effects between SNPs in these five genes and NAFLD-related quantitative traits, T2D, central obesity, and MetS.

## Supporting Information

Supporting Information S1 **Summary of the number of abstracts retrieved from PubMed using different search phrases.**
(DOC)
