# Supplementary material for: Bioinformatics-Driven Identification and Examination of Candidate Genes for Non-Alcoholic Fatty Liver Disease
Source: PLoS One. 2011 Jan 27;6(1):e16542. doi: 10.1371/journal.pone.0016542 (PMC3029374; doi:10.1371/journal.pone.0016542)
Supplement: Supporting Information S1 — Summary of the number of abstracts retrieved from PubMed using different search phrases. (DOC) [file pone.0016542.s001.doc]

**Supporting information S1**

Summary of the number of abstracts retrieved from PubMed using different search phrases

| ***Search phrase*** | ***Number of abstracts*** |
| --- | --- |
| “NAFLD” | 876 |
| “non-alcoholic fatty liver disease” | 523 (661[[1]](#footnote-2)) |
| “hepatic steatosis” | 1062 |
| **(“hepatic steatosis” OR “NAFLD”) AND “genes”** | **172** |
| **(“visceral obesity” OR “waist circumference”) AND “genes”** | **147** |
| (“hepatic steatosis” OR “NAFLD”) AND “gene” | 277 |
| (“visceral obesity” OR “waist circumference”) AND “gene” | 270 |

The search phrases used in this study are highlighted in bold.

Limits: published within the years 2003-2008 and in English.

**Scoring scheme for prioritizing genes from the text curation analysis based on the amount of supporting data**

|  | **Expression studies** | | **Rodent models** | | **Cell studies** | | **Genetic association studies** | | **OMIM phenotypes** | |  |  |
| --- | --- | --- | --- | --- | --- | --- | --- | --- | --- | --- | --- | --- |
| **Gene** | **Score = 1** | | **Score = 1** | | **Score = 1** | | **Score = 2** | | **Score = 1 or 2** | | **Total score** | **References** |
| ABCA1 |  |  | ** | 1 |  |  |  |  |  |  | 1 |  |
| ABCB11 |  |  | * | 1 |  |  |  |  |  |  | 1 |  |
| ABR | * | 1 |  |  |  |  |  |  |  |  | 1 |  |
| ACADM |  |  |  |  |  |  |  |  | ++ | 2 | 2 |  |
| ACADSB | ** | 1 |  |  |  |  |  |  |  |  | 1 |  |
| ACAT1/MAT1A |  |  | * | 1 |  |  |  |  |  |  | 1 |  |
| ACAT2 | * | 1 |  |  |  |  |  |  |  |  | 1 |  |
| ACC1 | ** | 1 | *** | 1 |  |  |  |  |  |  | 2 |  |
| ACC2 | ** | 1 |  |  |  |  |  |  |  |  | 1 |  |
| ACL |  |  | * | 1 |  |  |  |  |  |  | 1 |  |
| ACOX | * | 1 |  |  |  |  |  |  | + | 1 | 2 |  |
| ACSL4 | ** | 1 |  |  |  |  |  |  |  |  | 1 |  |
| ACSL5 |  |  | * | 1 |  |  |  |  |  |  | 1 |  |
| ACTG1 |  |  |  |  |  |  |  |  |  |  | 0 |  |
| ACTR2 | * | 1 |  |  |  |  |  |  |  |  | 1 |  |
| ADAMTS1 |  |  |  |  |  |  |  |  |  |  | 0 |  |
| ADFP |  |  | * | 1 |  |  |  |  |  |  | 1 |  |
| ADH/ALDH |  |  |  |  |  |  |  |  |  |  | 0 |  |
| ADIPONECTIN |  |  | * | 1 |  |  | * | 2 |  |  | 3 |  |
| ADK |  |  |  |  |  |  |  |  | + | 1 | 1 |  |
| ADM | * | 1 |  |  |  |  |  |  |  |  | 1 |  |
| ADRB2 |  |  |  |  |  |  | * | 2 |  |  | 2 |  |
| AFP | * | 1 |  |  |  |  |  |  |  |  | 1 |  |
| AGPAT2 |  |  |  |  |  |  |  |  | ++ | 2 | 2 |  |
| AGT |  |  |  |  |  |  |  |  |  |  | 0 |  |
| AHSG |  |  | * | 1 |  |  |  |  |  |  | 1 |  |
| AKR1C1 | * | 1 |  |  |  |  |  |  |  |  | 1 |  |
| AKR1C2 | * | 1 |  |  |  |  |  |  |  |  | 1 |  |
| AKT2 |  |  |  |  |  |  |  |  |  |  | 0 |  |
| ALAS | * | 1 |  |  |  |  |  |  |  |  | 1 |  |
| ALDH1B1 | * | 1 | * | 1 |  |  |  |  |  |  | 2 |  |
| ALMS1 |  |  |  |  |  |  |  |  | ++ | 2 | 2 |  |
| APOA1 |  |  | * | 1 |  |  |  |  |  |  | 1 |  |
| APOA5 |  |  |  |  |  |  |  |  |  |  | 0 |  |
| APOB |  |  |  |  |  |  |  |  |  |  | 0 |  |
| APOC4 |  |  |  |  |  |  |  |  |  |  | 0 |  |
| APOE |  |  |  |  |  |  |  |  |  |  | 0 |  |
| APOL3 | * | 1 |  |  |  |  |  |  |  |  | 1 |  |
| APOM | * | 1 |  |  |  |  |  |  |  |  | 1 |  |
| ARG1 |  |  | * | 1 |  |  |  |  |  |  | 1 |  |
| ARL6IP/AIP1/ARMER | ** | 1 |  |  |  |  |  |  |  |  | 1 |  |
| ASL |  |  | * | 1 |  |  |  |  |  |  | 1 |  |
| ASNS | * | 1 |  |  |  |  |  |  |  |  | 1 |  |
| ASS1 |  |  | * | 1 |  |  |  |  |  |  | 1 |  |
| ATF3 | * | 1 |  |  |  |  |  |  |  |  | 1 |  |
| ATGL |  |  | * | 1 |  |  |  |  |  |  | 1 |  |
| BCAT1 |  |  |  |  |  |  |  |  |  |  | 0 |  |
| BDNF |  |  | * | 1 |  |  |  |  |  |  | 1 |  |
| BSCL2 |  |  |  |  |  |  |  |  | ++ | 2 | 2 |  |
| C/EBPbeta |  |  | * | 1 |  |  |  |  |  |  | 1 |  |
| c12ORF14 | ** | 1 |  |  |  |  |  |  |  |  | 1 |  |
| C4BP |  |  | * | 1 |  |  |  |  |  |  | 1 |  |
| C5AR1 |  |  | * | 1 |  |  |  |  |  |  | 1 |  |
| C6 |  |  | * | 1 |  |  |  |  |  |  | 1 |  |
| CAR |  |  | * | 1 |  |  |  |  |  |  | 1 |  |
| CASP8 |  |  | * | 1 |  |  |  |  |  |  | 1 |  |
| CAT | * | 1 |  |  |  |  |  |  |  |  | 1 |  |
| CCL2 |  |  |  |  |  |  |  |  |  |  | 0 |  |
| CCL4 |  |  |  |  |  |  |  |  |  |  | 0 |  |
| CCL7/MCP-3 | * | 1 |  |  |  |  |  |  |  |  | 1 |  |
| CCND1 |  |  | * | 1 |  |  |  |  |  |  | 1 |  |
| CD14 |  |  |  |  |  |  |  |  |  |  | 0 |  |
| CD36 | * | 1 | ** | 1 |  |  |  |  |  |  | 2 |  |
| CDSP |  |  |  |  |  |  |  |  | ++ | 2 | 2 |  |
| CGL2 |  |  |  |  |  |  |  |  |  |  | 0 |  |
| CHI3L1 | * | 1 |  |  |  |  |  |  |  |  | 1 |  |
| ChREBP |  |  | ** | 1 |  |  |  |  |  |  | 1 |  |
| CMKOR1 |  |  |  |  |  |  |  |  |  |  | 0 |  |
| CNR1 |  |  |  |  |  |  | * | 1 |  |  | 1 |  |
| COL15A1 |  |  |  |  |  |  |  |  |  |  | 0 |  |
| COL2IA1 |  |  |  |  |  |  |  |  |  |  | 0 |  |
| COL4A3 | * | 1 |  |  |  |  |  |  |  |  | 1 |  |
| COL7A1 |  |  |  |  |  |  |  |  |  |  | 0 |  |
| CPT1/CPT1a | ** | 1 | * | 1 |  |  |  |  |  |  | 2 |  |
| CSPG2 | ** | 1 |  |  |  |  |  |  |  |  | 1 |  |
| CSTA | * | 1 |  |  |  |  |  |  |  |  | 1 |  |
| CTGF |  |  |  |  |  |  |  |  |  |  | 0 |  |
| CTHRC1 |  |  |  |  |  |  |  |  |  |  | 0 |  |
| CTLA-4 |  |  |  |  |  |  |  |  |  |  | 0 |  |
| CTNNB1 |  |  |  |  |  |  |  |  |  |  | 0 |  |
| CUTL2 | * | 1 |  |  |  |  |  |  |  |  | 1 |  |
| CYP2E1 | ** | 1 |  |  |  |  |  |  |  |  | 1 |  |
| CYP4 |  |  |  |  |  |  |  |  |  |  | 0 |  |
| CYP51A1 | * | 1 |  |  |  |  |  |  |  |  | 1 |  |
| CYP7A1 | * | 1 |  |  |  |  |  |  |  |  | 1 |  |
| CYP7B1 |  |  | * | 1 |  |  |  |  |  |  | 1 |  |
| DDX5 |  |  |  |  |  |  |  |  |  |  | 0 |  |
| DGAT1 | ** | 1 | *** | 1 |  |  |  |  |  |  | 2 |  |
| DGAT2 |  |  | ** | 1 |  |  |  |  |  |  | 1 |  |
| DHCR7 | * | 1 |  |  |  |  |  |  |  |  | 1 |  |
| dj462o23.2 | ** | 1 |  |  |  |  |  |  |  |  | 1 |  |
| DLD |  |  |  |  |  |  |  |  |  |  | 0 |  |
| DNAJC19 |  |  |  |  |  |  |  |  | ++ | 2 | 2 |  |
| EDIL3 | * | 1 |  |  |  |  |  |  |  |  | 1 |  |
| ELOVL2 |  |  |  |  |  |  |  |  |  |  | 0 |  |
| ELOVL3 |  |  | * | 1 |  |  |  |  |  |  | 1 |  |
| Epas1 |  |  | * | 1 |  |  |  |  |  |  | 1 |  |
| ESR1 |  |  |  |  |  |  | * | 1 |  |  | 1 |  |
| ETS2 | * | 1 |  |  |  |  |  |  |  |  | 1 |  |
| F13A1 |  |  |  |  |  |  |  |  |  |  | 0 |  |
| FABP2 |  |  |  |  |  |  | * | 1 |  |  | 1 |  |
| FABP4 |  |  |  |  |  |  |  |  |  |  | 0 |  |
| FABP5 |  |  | * | 1 |  |  |  |  |  |  | 1 |  |
| FADS2 |  |  | * | 1 |  |  |  |  |  |  | 1 |  |
| FAS | ** | 1 | ******* | 1 |  |  |  |  |  |  | 2 |  |
| FATP |  |  | * | 1 |  |  |  |  |  |  | 1 |  |
| FGL1 | * | 1 |  |  |  |  |  |  |  |  | 1 |  |
| FIGF | ** | 1 |  |  |  |  |  |  |  |  | 1 |  |
| FLRT2 |  |  |  |  |  |  |  |  |  |  | 0 |  |
| GCCR |  |  |  |  |  |  |  |  |  |  | 0 |  |
| GCK |  |  |  |  |  |  |  |  |  |  | 0 |  |
| GDAP1 |  |  |  |  |  |  |  |  |  |  | 0 |  |
| GH |  |  |  |  |  |  |  |  | + | 1 | 1 |  |
| GLIPR1 | * | 1 |  |  |  |  |  |  |  |  | 1 |  |
| GLP-1 |  |  | * | 1 |  |  |  |  |  |  | 1 |  |
| GLUT1 |  |  | * | 1 |  |  |  |  |  |  | 1 |  |
| GLUT4 |  |  | * | 1 |  |  |  |  |  |  | 1 |  |
| GOT1 | * | 1 |  |  |  |  |  |  |  |  | 1 |  |
| GP |  |  | * | 1 |  |  |  |  |  |  | 1 |  |
| GPD2 | * | 1 | * | 1 |  |  |  |  |  |  | 2 |  |
| GPI |  |  |  |  |  |  |  |  |  |  | 0 |  |
| GPI-PLD | * | 1 |  |  |  |  |  |  |  |  | 1 |  |
| GSS | * | 1 |  |  |  |  |  |  |  |  | 1 |  |
| GST |  |  |  |  |  |  |  |  |  |  | 0 |  |
| GSTA1 |  |  | * | 1 |  |  |  |  |  |  | 1 |  |
| GSTA2 |  |  | * | 1 |  |  |  |  |  |  | 1 |  |
| GSTA4 | * | 1 |  |  |  |  |  |  |  |  | 1 |  |
| GSTM1 |  |  | * | 1 |  |  |  |  |  |  | 1 |  |
| GSTO1 |  |  | * | 1 |  |  |  |  |  |  | 1 |  |
| GSTT2 |  |  | * | 1 |  |  |  |  |  |  | 1 |  |
| GYG2 | * | 1 |  |  |  |  |  |  |  |  | 1 |  |
| HDAC1 |  |  | * | 1 |  |  |  |  |  |  | 1 |  |
| HDL |  |  |  |  |  |  |  |  | + | 1 | 1 |  |
| HFE |  |  |  |  |  |  |  |  |  |  | 0 |  |
| HK1 | * | 1 |  |  |  |  |  |  |  |  | 1 |  |
| HMGCR | * | 1 | * | 1 |  |  |  |  |  |  | 2 |  |
| HMGCS1 |  |  | * | 1 |  |  |  |  |  |  | 1 |  |
| HMGCS2 | ** | 1 |  |  |  |  |  |  |  |  | 1 |  |
| HNF4 |  |  | * | 1 |  |  |  |  |  |  | 1 |  |
| hnRNPK | * | 1 |  |  |  |  |  |  |  |  | 1 |  |
| HSD17B12 |  |  | * | 1 |  |  |  |  |  |  | 1 |  |
| HSL (LIPE) | ** | 1 | * | 1 |  |  | * | 2 |  |  | 4 |  |
| IDI1 | * | 1 |  |  |  |  |  |  |  |  | 1 |  |
| IFNA7 |  |  |  |  |  |  |  |  |  |  | 0 |  |
| IFNgamma | * | 1 |  |  |  |  |  |  |  |  | 1 |  |
| IGFBP1 | * | 1 |  |  |  |  |  |  |  |  | 1 |  |
| IGFBP2 | * | 1 |  |  |  |  |  |  |  |  | 1 |  |
| IKBKG |  |  | * | 1 |  |  |  |  |  |  | 1 |  |
| IL-10 |  |  |  |  |  |  |  |  |  |  | 0 |  |
| IL-18 |  |  |  |  |  |  |  |  |  |  | 0 |  |
| IL-1RN | ** | 1 |  |  |  |  |  |  |  |  | 1 |  |
| IL-4 |  |  |  |  |  |  |  |  |  |  | 0 |  |
| IL-6 |  |  | * | 1 |  |  |  |  |  |  | 1 |  |
| IL7R |  |  |  |  |  |  |  |  |  |  | 0 |  |
| INSIG1 | * | 1 |  |  |  |  |  |  |  |  | 1 |  |
| INSR |  |  |  |  |  |  |  |  |  |  | 0 |  |
| IRS | * | 1 | * | 1 |  |  |  |  |  |  | 2 |  |
| ITGA9 |  |  |  |  |  |  |  |  |  |  | 0 |  |
| JUN | ** | 1 |  |  |  |  |  |  |  |  | 1 |  |
| KCNN2 |  |  |  |  |  |  |  |  |  |  | 0 |  |
| KIAA1333 | * | 1 |  |  |  |  |  |  |  |  | 1 |  |
| LAMA1 |  |  |  |  |  |  |  |  |  |  | 0 |  |
| LAMA4 |  |  |  |  |  |  |  |  |  |  | 0 |  |
| LCAD | ** | 1 | * | 1 |  |  |  |  | + | 1 | 3 |  |
| LCHAD |  |  |  |  |  |  |  |  | ++ | 2 | 2 |  |
| LDLR |  |  |  |  |  |  |  |  |  |  | 0 |  |
| LEPTIN |  |  | * | 1 |  |  |  |  |  |  | 1 |  |
| L-FAB |  |  | ** | 1 |  |  |  |  |  |  | 1 |  |
| LIFR |  |  | * | 1 |  |  |  |  |  |  | 1 |  |
| LIPC |  |  |  |  |  |  |  |  | + | 1 | 1 |  |
| LMNA |  |  |  |  |  |  |  |  | ++ | 2 | 2 |  |
| LPAL2 | * | 1 |  |  |  |  |  |  |  |  | 1 |  |
| LTF | * | 1 |  |  |  |  |  |  |  |  | 1 |  |
| LXRα |  |  | ** | 1 | * | 1 |  |  |  |  | 2 |  |
| LXRβ |  |  | ** | 1 |  |  |  |  |  |  | 1 |  |
| MAP2K4 |  |  |  |  |  |  |  |  |  |  | 0 |  |
| MAPK11 |  |  | * | 1 |  |  |  |  |  |  | 1 |  |
| MC4R |  |  | * | 1 |  |  |  |  |  |  | 1 |  |
| MCH1 |  |  | * | 1 |  |  |  |  |  |  | 1 |  |
| ME2 | * | 1 |  |  |  |  |  |  |  |  | 1 |  |
| ME3 | * | 1 |  |  |  |  |  |  |  |  | 1 |  |
| MGC4504 | * | 1 |  |  |  |  |  |  |  |  | 1 |  |
| MGLL |  |  | * | 1 |  |  |  |  |  |  | 1 |  |
| MMACHC |  |  |  |  |  |  |  |  | ++ | 2 | 2 |  |
| MMP |  |  |  |  |  |  |  |  |  |  | 0 |  |
| MMP15 |  |  |  |  |  |  |  |  |  |  | 0 |  |
| MMP2 | ** | 1 |  |  |  |  |  |  |  |  | 1 |  |
| MMP7 |  |  |  |  |  |  |  |  |  |  | 0 |  |
| MT1 |  |  | * | 1 |  |  |  |  |  |  | 1 |  |
| MT1X | * | 1 |  |  |  |  |  |  |  |  | 1 |  |
| MT2A | * | 1 |  |  |  |  |  |  |  |  | 1 |  |
| MTCYB |  |  |  |  |  |  |  |  | + | 1 | 1 |  |
| MTHFR |  |  |  |  |  |  | * | 2 |  |  | 2 |  |
| MTTP |  |  |  |  |  |  | * | 2 |  |  | 2 |  |
| MYLIP | * | 1 |  |  |  |  |  |  |  |  | 1 |  |
| NFIL-3 | * | 1 |  |  |  |  |  |  |  |  | 1 |  |
| NNMT | * | 1 |  |  |  |  |  |  |  |  | 1 |  |
| NOCTURNIN |  |  | * | 1 |  |  |  |  |  |  | 1 |  |
| NOD2 |  |  |  |  |  |  |  |  |  |  | 0 |  |
| NOX4 |  |  | * | 1 |  |  |  |  |  |  | 1 |  |
| NR2F2/COUP-TFII | * | 1 |  |  |  |  |  |  |  |  | 1 |  |
| Nrf-1 |  |  |  |  |  |  |  |  |  |  | 0 |  |
| OAT | ** | 1 |  |  |  |  |  |  |  |  | 1 |  |
| PAPPA | * | 1 |  |  |  |  |  |  |  |  | 1 |  |
| PDHA1 |  |  |  |  |  |  |  |  |  |  | 0 |  |
| PEG10 | * | 1 |  |  |  |  |  |  |  |  | 1 |  |
| PEMT |  |  |  |  |  |  |  |  |  |  | 0 |  |
| PEPCK |  |  | * | 1 |  |  |  |  |  |  | 1 |  |
| PGM3 |  |  |  |  |  |  |  |  |  |  | 0 |  |
| PHKA2 |  |  |  |  |  |  |  |  |  |  | 0 |  |
| PITPalpha |  |  | * | 1 |  |  |  |  |  |  | 1 |  |
| PLIN |  |  |  |  |  |  |  |  |  |  | 0 |  |
| PLSCR1 | * | 1 |  |  |  |  |  |  |  |  | 1 |  |
| PNRC1 | * | 1 |  |  |  |  |  |  |  |  | 1 |  |
| POU2AF1 | ** | 1 |  |  |  |  |  |  |  |  | 1 |  |
| PPARc |  |  | * | 1 |  |  |  |  |  |  | 1 |  |
| PPARα | ** | 1 | ********** | 1 |  |  |  |  |  |  | 2 |  |
| PPARγ | ** | 1 | ***** | 1 |  |  | ** | 2 |  |  | 4 |  |
| PPP1R1A |  |  |  |  |  |  |  |  |  |  | 0 |  |
| PPP1R2 |  |  |  |  |  |  |  |  |  |  | 0 |  |
| PRSS3 | ** | 1 |  |  |  |  |  |  |  |  | 1 |  |
| PTLP |  |  |  |  |  |  | * | 2 |  |  | 2 |  |
| PTPRF |  |  |  |  |  |  |  |  |  |  | 0 |  |
| PTX3 | * | 1 |  |  |  |  |  |  |  |  | 1 |  |
| PYGM |  |  |  |  |  |  |  |  |  |  | 0 |  |
| RAR |  |  |  |  |  |  |  |  |  |  | 0 |  |
| RESISTIN |  |  | * | 1 |  |  | * | 2 |  |  | 3 |  |
| RIP140 |  |  | * | 1 |  |  |  |  |  |  | 1 |  |
| ROD1 | ** | 1 |  |  |  |  |  |  |  |  | 1 |  |
| S100A8 | * | 1 |  |  |  |  |  |  |  |  | 1 |  |
| SC5D |  |  | * | 1 |  |  |  |  |  |  | 1 |  |
| SCARB1 |  |  | * | 1 |  |  |  |  |  |  | 1 |  |
| SCD | * | 1 | ******* | 1 |  |  | * | 1 |  |  | 3 |  |
| SDC4 | * | 1 |  |  |  |  |  |  |  |  | 1 |  |
| SDS | ** | 1 |  |  |  |  |  |  |  |  | 1 |  |
| SEC8LI |  |  |  |  |  |  |  |  |  |  | 0 |  |
| SERPINA3 | * | 1 |  |  |  |  |  |  |  |  | 1 |  |
| SERPINB5 |  |  | * | 1 |  |  |  |  |  |  | 1 |  |
| SERPINE2 | * | 1 |  |  |  |  |  |  |  |  | 1 |  |
| SGK | * | 1 |  |  |  |  |  |  |  |  | 1 |  |
| SH3BGRL2 | * | 1 |  |  |  |  |  |  |  |  | 1 |  |
| SLC35B4 |  |  |  |  |  |  |  |  |  |  | 0 |  |
| SOCS1 | * | 1 | * | 1 |  |  |  |  |  |  | 2 |  |
| SOCS3 |  |  | * | 1 |  |  |  |  |  |  | 1 |  |
| SOD | * | 1 |  |  |  |  |  |  |  |  | 1 |  |
| SOD2 |  |  |  |  |  |  |  |  |  |  | 0 |  |
| SOD3 |  |  |  |  |  |  |  |  |  |  | 0 |  |
| SPARCL1 |  |  |  |  |  |  |  |  |  |  | 0 |  |
| SQLE |  |  | * | 1 |  |  |  |  |  |  | 1 |  |
| SQLE | * | 1 |  |  |  |  |  |  |  |  | 1 |  |
| SSTR2 |  |  |  |  |  |  |  |  |  |  | 0 |  |
| STXBP1 | * | 1 |  |  |  |  |  |  |  |  | 1 |  |
| SULT1A2 | * | 1 |  |  |  |  |  |  |  |  | 1 |  |
| SAA4 |  |  | * | 1 |  |  |  |  |  |  | 1 |  |
| TBC1D1 |  |  |  |  |  |  |  |  |  |  | 0 |  |
| TF | * | 1 |  |  |  |  |  |  |  |  | 1 |  |
| TGFB1 |  |  |  |  |  |  |  |  |  |  | 0 |  |
| TGFβ | * | 1 | * | 1 |  |  |  |  |  |  | 2 |  |
| THBS1 | * | 1 |  |  |  |  |  |  |  |  | 1 |  |
| TIMP |  |  |  |  |  |  |  |  |  |  | 0 |  |
| TLR4 |  |  |  |  |  |  |  |  |  |  | 0 |  |
| TNFRSF17 |  |  |  |  |  |  |  |  |  |  | 0 |  |
| TNFα | * | 1 | ** | 1 |  |  |  |  |  |  | 2 |  |
| UCP |  |  | ** | 1 |  |  |  |  |  |  | 1 |  |
| UCP2 | ** | 1 | * | 1 |  |  |  |  |  |  | 2 |  |
| UGCG |  |  |  |  |  |  |  |  |  |  | 0 |  |
| VLCAD |  |  | * | 1 |  |  |  |  | ++ | 2 | 3 |  |
| VLDLR |  |  |  |  |  |  |  |  |  |  | 0 |  |
| ZFP36 | * | 1 |  |  |  |  |  |  |  |  | 1 |  |
| ZMPSTE24 |  |  |  |  |  |  |  |  |  |  | 0 |  |

If a gene was mentioned in context with NAFLD in 1) an expression study i.e. a microarray study, or 2) a study of rodents with NAFLD, or 3) cell studies investigating NAFLD, or 4) genetic association studies of humans with NAFLD, the gene was assigned a *. For each data source a * were equal to a score of either 1 (data source 1-3) or 2 (data source 4). Similarly, OMIM was mined for genetic abnormalities leading to syndromes with NAFLD-related phenotypes. Genes were assigned a score of 2 (++) if directly associated with NAFLD in an OMIM record, and 1 (+) if indirectly associated with NAFLD-related phenotypes. Genes in the rows highlighted in gray are the genes prioritized for bioinformatics analysis. References are included below.

References:

1. Miao B, Zondlo S, Gibbs S, Cromley D, Hosagrahara VP, et al. (2004) Raising HDL cholesterol without inducing hepatic steatosis and hypertriglyceridemia by a selective LXR modulator. J Lipid Res 45: 1410-1417.

2. Toye AA, Dumas ME, Blancher C, Rothwell AR, Fearnside JF, et al. (2007) Subtle metabolic and liver gene transcriptional changes underlie diet-induced fatty liver susceptibility in insulin-resistant mice. Diabetologia 50: 1867-1879.

3. Figge A, Lammert F, Paigen B, Henkel A, Matern S, et al. (2004) Hepatic overexpression of murine Abcb11 increases hepatobiliary lipid secretion and reduces hepatic steatosis. J Biol Chem 279: 2790-2799.

4. Younossi ZM, Gorreta F, Ong JP, Schlauch K, Del Giacco L, et al. (2005) Hepatic gene expression in patients with obesity-related non-alcoholic steatohepatitis. Liver Int 25: 760-771.

5. Greco D, Kotronen A, Westerbacka J, Puig O, Arkkila P, et al. (2008) Gene expression in human NAFLD. Am J Physiol Gastrointest Liver Physiol 294: G1281-1287.

6. Younossi ZM, Baranova A, Ziegler K, Del Giacco L, Schlauch K, et al. (2005) A genomic and proteomic study of the spectrum of nonalcoholic fatty liver disease. Hepatology 42: 665-674.

7. Wilfred de Alwis NM, Day CP (2008) Genes and nonalcoholic fatty liver disease. CurrDiabRep 8: 156-163.

8. Wilfred de Alwis NM, Day CP (2007) Genetics of alcoholic liver disease and nonalcoholic fatty liver disease. Semin Liver Dis 27: 44-54.

9. De Gottardi A, Vinciguerra M, Sgroi A, Moukil M, Ravier-Dall'Antonia F, et al. (2007) Microarray analyses and molecular profiling of steatosis induction in immortalized human hepatocytes. Lab Invest 87: 792-806.

10. Kohjima M, Enjoji M, Higuchi N, Kato M, Kotoh K, et al. (2007) Re-evaluation of fatty acid metabolism-related gene expression in nonalcoholic fatty liver disease. Int J Mol Med 20: 351-358.

11. Nakamuta M, Kohjima M, Morizono S, Kotoh K, Yoshimoto T, et al. (2005) Evaluation of fatty acid metabolism-related gene expression in nonalcoholic fatty liver disease. Int J Mol Med 16: 631-635.

12. Heijboer AC, Donga E, Voshol PJ, Dang ZC, Havekes LM, et al. (2005) Sixteen hours of fasting differentially affects hepatic and muscle insulin sensitivity in mice. J Lipid Res 46: 582-588.

13. Choi CS, Savage DB, Kulkarni A, Yu XX, Liu ZX, et al. (2007) Suppression of diacylglycerol acyltransferase-2 (DGAT2), but not DGAT1, with antisense oligonucleotides reverses diet-induced hepatic steatosis and insulin resistance. J Biol Chem 282: 22678-22688.

14. Kim HJ, Lee KT, Park YB, Jeon SM, Choi MS (2008) Dietary docosahexaenoic acid-rich diacylglycerols ameliorate hepatic steatosis and alter hepatic gene expressions in C57BL/6J-Lep(ob/ob) mice. Mol Nutr Food Res 52: 965-973.

15. Baranova A, Schlauch K, Elariny H, Jarrar M, Bennett C, et al. (2007) Gene expression patterns in hepatic tissue and visceral adipose tissue of patients with non-alcoholic fatty liver disease. Obes Surg 17: 1111-1118.

16. Berthier MT, Houde A, Cote M, Paradis AM, Mauriege P, et al. (2005) Impact of adiponectin gene polymorphisms on plasma lipoprotein and adiponectin concentrations of viscerally obese men. J Lipid Res 46: 237-244.

17. Begriche K, Letteron P, Abbey-Toby A, Vadrot N, Robin MA, et al. (2008) Partial leptin deficiency favors diet-induced obesity and related metabolic disorders in mice. Am J Physiol Endocrinol Metab 294: E939-951.

18. Mattevi VS, Zembrzuski VM, Hutz MH (2006) Impact of variation in ADRB2, ADRB3, and GNB3 genes on body mass index and waist circumference in a Brazilian population. Am J Hum Biol 18: 182-186.

19. Agarwal AK, Garg A (2006) Genetic disorders of adipose tissue development, differentiation, and death. Annu Rev Genomics Hum Genet 7: 175-199.

20. Day CP (2006) Genes or environment to determine alcoholic liver disease and non-alcoholic fatty liver disease. Liver Int 26: 1021-1028.

21. Hennige AM, Staiger H, Wicke C, Machicao F, Fritsche A, et al. (2008) Fetuin-A induces cytokine expression and suppresses adiponectin production. PLoS ONE 3: e1765.

22. Reid BN, Ables GP, Otlivanchik OA, Schoiswohl G, Zechner R, et al. (2008) Hepatic overexpression of hormone-sensitive lipase and adipose triglyceride lipase promotes fatty acid oxidation, stimulates direct release of free fatty acids, and ameliorates steatosis. J Biol Chem 283: 13087-13099.

23. Sha H, Xu J, Tang J, Ding J, Gong J, et al. (2007) Disruption of a novel regulatory locus results in decreased Bdnf expression, obesity, and type 2 diabetes in mice. Physiol Genomics 31: 252-263.

24. Rahman SM, Schroeder-Gloeckler JM, Janssen RC, Jiang H, Qadri I, et al. (2007) CCAAT/enhancing binding protein beta deletion in mice attenuates inflammation, endoplasmic reticulum stress, and lipid accumulation in diet-induced nonalcoholic steatohepatitis. Hepatology 45: 1108-1117.

25. Baskin-Bey ES, Anan A, Isomoto H, Bronk SF, Gores GJ (2007) Constitutive androstane receptor agonist, TCPOBOP, attenuates steatohepatitis in the methionine choline-deficient diet-fed mouse. World J Gastroenterol 13: 5635-5641.

26. Wang C, Pattabiraman N, Zhou JN, Fu M, Sakamaki T, et al. (2003) Cyclin D1 repression of peroxisome proliferator-activated receptor gamma expression and transactivation. Mol Cell Biol 23: 6159-6173.

27. Zhou J, Febbraio M, Wada T, Zhai Y, Kuruba R, et al. (2008) Hepatic fatty acid transporter Cd36 is a common target of LXR, PXR, and PPARgamma in promoting steatosis. Gastroenterology 134: 556-567.

28. Dentin R, Benhamed F, Hainault I, Fauveau V, Foufelle F, et al. (2006) Liver-specific inhibition of ChREBP improves hepatic steatosis and insulin resistance in ob/ob mice. Diabetes 55: 2159-2170.

29. Letexier D, Peroni O, Pinteur C, Beylot M (2005) In vivo expression of carbohydrate responsive element binding protein in lean and obese rats. Diabetes Metab 31: 558-566.

30. Russo P, Strazzullo P, Cappuccio FP, Tregouet DA, Lauria F, et al. (2007) Genetic variations at the endocannabinoid type 1 receptor gene (CNR1) are associated with obesity phenotypes in men. J Clin Endocrinol Metab 92: 2382-2386.

31. Fox CS, Heard-Costa N, Cupples LA, Dupuis J, Vasan RS, et al. (2007) Genome-wide association to body mass index and waist circumference: the Framingham Heart Study 100K project. BMC Med Genet 8 Suppl 1: S18.

32. Hardwick JP (2008) Cytochrome P450 omega hydroxylase (CYP4) function in fatty acid metabolism and metabolic diseases. Biochem Pharmacol 75: 2263-2275.

33. Yu XX, Murray SF, Pandey SK, Booten SL, Bao D, et al. (2005) Antisense oligonucleotide reduction of DGAT2 expression improves hepatic steatosis and hyperlipidemia in obese mice. Hepatology 42: 362-371.

34. Scortegagna M, Ding K, Oktay Y, Gaur A, Thurmond F, et al. (2003) Multiple organ pathology, metabolic abnormalities and impaired homeostasis of reactive oxygen species in Epas1-/- mice. Nat Genet 35: 331-340.

35. Gallagher CJ, Langefeld CD, Gordon CJ, Campbell JK, Mychaleckyj JC, et al. (2007) Association of the estrogen receptor-alpha gene with the metabolic syndrome and its component traits in African-American families: the Insulin Resistance Atherosclerosis Family Study. Diabetes 56: 2135-2141.

36. Martinez-Lopez E, Ruiz-Madrigal B, Hernandez-Canaveral I, Panduro A (2007) Association of the T54 allele of the FABP2 gene with cardiovascular risk factors in obese Mexican subjects. Diab Vasc Dis Res 4: 235-236.

37. Newberry EP, Xie Y, Kennedy SM, Luo J, Davidson NO (2006) Protection against Western diet-induced obesity and hepatic steatosis in liver fatty acid-binding protein knockout mice. Hepatology 44: 1191-1205.

38. Rinella ME, Elias MS, Smolak RR, Fu T, Borensztajn J, et al. (2008) Mechanisms of hepatic steatosis in mice fed a lipogenic methionine choline-deficient diet. J Lipid Res 49: 1068-1076.

39. Ding X, Saxena NK, Lin S, Gupta NA, Anania FA (2006) Exendin-4, a glucagon-like protein-1 (GLP-1) receptor agonist, reverses hepatic steatosis in ob/ob mice. Hepatology 43: 173-181.

40. Emanuelli B, Eberle D, Suzuki R, Kahn CR (2008) Overexpression of the dual-specificity phosphatase MKP-4/DUSP-9 protects against stress-induced insulin resistance. Proc Natl Acad Sci U S A 105: 3545-3550.

41. Chalasani N, Vuppalanchi R, Raikwar NS, Deeg MA (2006) Glycosylphosphatidylinositol-specific phospholipase d in nonalcoholic Fatty liver disease: a preliminary study. J Clin Endocrinol Metab 91: 2279-2285.

42. Wang AG, Seo SB, Moon HB, Shin HJ, Kim DH, et al. (2005) Hepatic steatosis in transgenic mice overexpressing human histone deacetylase 1. Biochem Biophys Res Commun 330: 461-466.

43. Bragoszewski P, Habior A, Walewska-Zielecka B, Ostrowski J (2007) Expression of genes encoding mitochondrial proteins can distinguish nonalcoholic steatosis from steatohepatitis. Acta Biochim Pol 54: 341-348.

44. van der Leij FR, Bloks VW, Grefhorst A, Hoekstra J, Gerding A, et al. (2007) Gene expression profiling in livers of mice after acute inhibition of beta-oxidation. Genomics 90: 680-689.

45. Goetzman ES, Tian L, Wood PA (2005) Differential induction of genes in liver and brown adipose tissue regulated by peroxisome proliferator-activated receptor-alpha during fasting and cold exposure in acyl-CoA dehydrogenase-deficient mice. Mol Genet Metab 84: 39-47.

46. Spann NJ, Kang S, Li AC, Chen AZ, Newberry EP, et al. (2006) Coordinate transcriptional repression of liver fatty acid-binding protein and microsomal triglyceride transfer protein blocks hepatic very low density lipoprotein secretion without hepatosteatosis. J Biol Chem 281: 33066-33077.

47. Kim KH, Choi SH, Lee TS, Oh WK, Kim DS, et al. (2006) Selective LXRalpha inhibitory effects observed in plant extracts of MEH184 (Parthenocissua tricuspidata) and MEH185 (Euscaphis japonica). Biochem Biophys Res Commun 349: 513-518.

48. Lund EG, Peterson LB, Adams AD, Lam MH, Burton CA, et al. (2006) Different roles of liver X receptor alpha and beta in lipid metabolism: effects of an alpha-selective and a dual agonist in mice deficient in each subtype. Biochem Pharmacol 71: 453-463.

49. Albarado DC, McClaine J, Stephens JM, Mynatt RL, Ye J, et al. (2004) Impaired coordination of nutrient intake and substrate oxidation in melanocortin-4 receptor knockout mice. Endocrinology 145: 243-252.

50. Gomori A, Ishihara A, Ito M, Matsushita H, Mashiko S, et al. (2007) Blockade of MCH1 receptor signalling ameliorates obesity and related hepatic steatosis in ovariectomized mice. Br J Pharmacol 151: 900-908.

51. Sazci A, Ergul E, Aygun C, Akpinar G, Senturk O, et al. (2008) Methylenetetrahydrofolate reductase gene polymorphisms in patients with nonalcoholic steatohepatitis (NASH). Cell Biochem Funct 26: 291-296.

52. Berthier MT, Houde A, Paradis AM, Couture P, Gaudet D, et al. (2004) Molecular screening of the microsomal triglyceride transfer protein: association between polymorphisms and both abdominal obesity and plasma apolipoprotein B concentration. J Hum Genet 49: 684-690.

53. Green CB, Douris N, Kojima S, Strayer CA, Fogerty J, et al. (2007) Loss of Nocturnin, a circadian deadenylase, confers resistance to hepatic steatosis and diet-induced obesity. Proc Natl Acad Sci U S A 104: 9888-9893.

54. Alb JG, Jr., Phillips SE, Wilfley LR, Philpot BD, Bankaitis VA (2007) The pathologies associated with functional titration of phosphatidylinositol transfer protein alpha activity in mice. J Lipid Res 48: 1857-1872.

55. Harano Y, Yasui K, Toyama T, Nakajima T, Mitsuyoshi H, et al. (2006) Fenofibrate, a peroxisome proliferator-activated receptor alpha agonist, reduces hepatic steatosis and lipid peroxidation in fatty liver Shionogi mice with hereditary fatty liver. Liver Int 26: 613-620.

56. Bosse Y, Bouchard L, Despres JP, Bouchard C, Perusse L, et al. (2005) Haplotypes in the phospholipid transfer protein gene are associated with obesity-related phenotypes: the Quebec Family Study. Int J Obes (Lond) 29: 1338-1345.

57. Leonardsson G, Steel JH, Christian M, Pocock V, Milligan S, et al. (2004) Nuclear receptor corepressor RIP140 regulates fat accumulation. Proc Natl Acad Sci U S A 101: 8437-8442.

58. Rizki G, Arnaboldi L, Gabrielli B, Yan J, Lee GS, et al. (2006) Mice fed a lipogenic methionine-choline-deficient diet develop hypermetabolism coincident with hepatic suppression of SCD-1. J Lipid Res 47: 2280-2290.

59. Sampath H, Ntambi JM (2006) Stearoyl-coenzyme A desaturase 1, sterol regulatory element binding protein-1c and peroxisome proliferator-activated receptor-alpha: independent and interactive roles in the regulation of lipid metabolism. Curr Opin Clin Nutr Metab Care 9: 84-88.

60. Ueki K, Kondo T, Tseng YH, Kahn CR (2004) Central role of suppressors of cytokine signaling proteins in hepatic steatosis, insulin resistance, and the metabolic syndrome in the mouse. Proc Natl Acad Sci U S A 101: 10422-10427.

61. Shirouchi B, Nagao K, Inoue N, Furuya K, Koga S, et al. (2008) Dietary phosphatidylinositol prevents the development of nonalcoholic fatty liver disease in Zucker (fa/fa) rats. J Agric Food Chem 56: 2375-2379.

62. Agarwal AK, Garg A (2006) Genetic basis of lipodystrophies and management of metabolic complications. Annu Rev Med 57: 297-311.

**LD-plots for the genotyped variants in each locus**


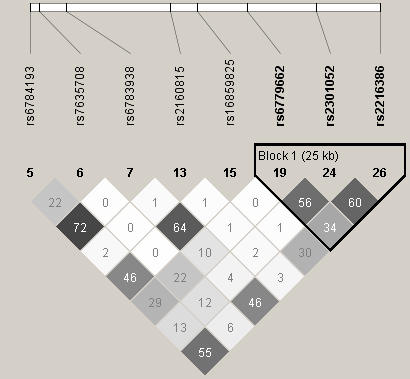

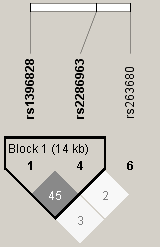

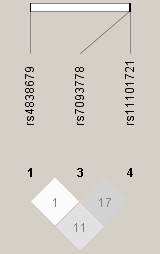

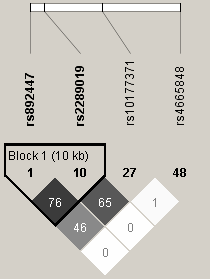


***EHHADH ECHS1 HADH***

***A/B ACADL***


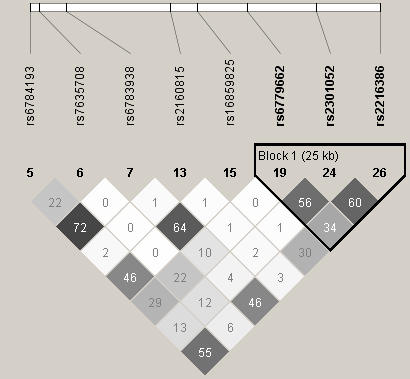

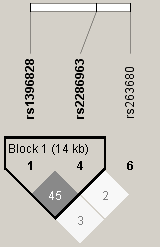

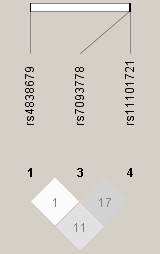

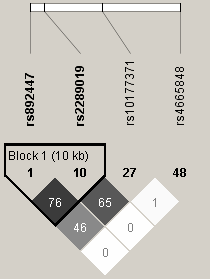


***EHHADH ECHS1 HADH***

***A/B ACADL***

LD displayed as r^2^ was generated in Haploview, using the r^2^ color scheme. The different shades of gray correspond to the r^2^ value in the square, dark being high LD and light being poor LD.

**Functional predictions for the variants**

|  |  |  | **Ensembl SNP Effect Predictor** | | | | **FastSNP** | | |
| --- | --- | --- | --- | --- | --- | --- | --- | --- | --- |
| **Variation** | **Major/minor  allele** | **Gene** | **Consequence** | **Position  (cDNA)** | **Position  (protein)** | **Amino acid  change** | **Possible Functional Effects** | **Lower risk** | **Upper risk** |
| rs4838679 | C/T | *ECHS1* | INTRONIC | N/A | N/A | N/A | Intronic with no known effect | 0 | 0 |
| rs7093778 | C/T | *ECHS1* | UPSTREAM | N/A | N/A | N/A | Promoter/regulatory region | 1 | 3 |
| rs11101721 | C/G | *ECHS1* | UPSTREAM | N/A | N/A | N/A | Promoter/regulatory region | 1 | 3 |
| rs1396828 | T/C | *ACADL* | WITHIN_NON_CODING_GENE | N/A | N/A | N/A | Intronic with no known effect | 0 | 0 |
| rs2286963 | T/G | *ACADL* | NON_SYNONYMOUS_CODING | 1225 | 333 | K/Q | Missense (coding) | 2 | 3 |
| rs892447 | G/A | *HADHA* | INTRONIC | N/A | N/A | N/A | Intronic enhancer | 1 | 2 |
| rs2289019 | G/C | *HADHA* | INTRONIC | N/A | N/A | N/A | Intronic with no known effect | 0 | 0 |
| rs10177371 | C/T | *HADHA* | INTRONIC | N/A | N/A | N/A | Promoter/intronic | 1 | 3 |
| rs4665848 | G/T | *HADHB* | INTERGENIC | N/A | N/A | N/A | *Not in db* | | |
| rs6784193 | A/G | *EHHADH* | DOWNSTREAM | N/A | N/A | N/A | Downstream with no known effect | 0 | 0 |
| rs7635708 | A/G | *EHHADH* | DOWNSTREAM | N/A | N/A | N/A | Intronic with no known effect | 0 | 0 |
| rs6783938 | C/T | *EHHADH* | DOWNSTREAM | N/A | N/A | N/A | Intronic with no known effect | 0 | 0 |
| rs2160815 | T/A | *EHHADH* | INTRONIC | N/A | N/A | N/A | Intronic with no known effect | 0 | 0 |
| rs16859825 | T/C | *EHHADH* | INTRONIC | N/A | N/A | N/A | Intronic with no known effect | 0 | 0 |
| rs6779662 | C/T | *EHHADH* | INTRONIC | N/A | N/A | N/A | Intronic with no known effect | 0 | 0 |
| rs2301052 | T/C | *EHHADH* | INTRONIC | N/A | N/A | N/A | Promoter/intronic | 1 | 3 |
| rs2216386 | G/A | *EHHADH* | INTRONIC | N/A | N/A | N/A | Intronic with no known effect | 0 | 0 |

The potential functional effects of the variants were predicted using Ensembl SNP Effect Predictor (http://www.ensembl.org/) and FastSNP .

FastSNP risk is defined as: 0, no effect; 1, very low effect; 2, low effect; 3, medium effect; 4, high effect; 5, very high effect.

Statistical power estimates

|  |  | Power | | |
| --- | --- | --- | --- | --- |
|  | MAF | 5 | 20 | 40 |
| **Quantitative trait analyses** | 0.5 cm change in waist circumference | 15% | 44% | 60% |
|  | 3% change in triglyceride levels | 32% | 76% | 91% |
|  | 0.03 mmol/l change in fasting plasma glucose | 44% | 93% | 98% |
|  | 5% change in fasting serum insulin | 48% | 96% | 99% |
| **Case-control analyses** |  |  |  |  |
| T2D | OR of 1.10 | 31% | 77% | 91% |
|  | OR of 1.15 | 59% | 98% | 100% |
|  | OR of 1.20 | 82% | 100% |  |
|  | OR of 1.25 | 95% |  |  |
| Obesity | OR of 1.10 | 48% | 93% | 99% |
|  | OR of 1.15 | 81% | 100% | 100% |
|  | OR of 1.20 | 96% |  |  |
|  | OR of 1.25 | 100% |  |  |
| MetS | OR of 1.10 | 18% | 48% | 64% |
|  | OR of 1.15 | 34% | 81% | 93% |
|  | OR of 1.20 | 54% | 96% | 99% |
|  | OR of 1.25 | 72% | 100% | 100% |

The statistical power calculations for quantitative traits were estimated in R using 1,000 simulations and a significance threshold of 0.05 in 6,162 individuals. The statistical power calculations in the case-control analyses were done using CaTS, power calculations for large genetic association studies, available at <http://www.sph.umich.edu/csg/abecasis/cats/>.

T2D: ~5,000 controls and ~3,500 T2D patients. Prevalence=0.08.

Obesity: ~5,800 controls and ~5,200 obese individuals. Prevalence=0.17 (Individuals with BMI≥30 from the Inter99).

MetS: ~1,750 controls and ~1,350 individuals with MetS. Prevalence (Inter99)=0.21

Genotype distribution and allele frequencies among glucose-tolerant control individuals and T2D patients

| Gene | SNP | Major/  minor | Controls/  Obese | n (men/women) | WT | HE | HO | MAF (%) | OR (95% CI) | *P_additive_* |
| --- | --- | --- | --- | --- | --- | --- | --- | --- | --- | --- |
| *EHHADH* | rs2216386 | A/G | Controls | 4855(2248/2607) | 3087(63.6) | 1572(32.4) | 196(4.0) | 20.2 (19.4-21.0) | 1.05(0.94-1.17) | 0.37 |
|  |  |  | T2D | 3452(2053/1399) | 2173(62.9) | 1135(32.9) | 144(4.2) | 20.6 (19.7-21.6) |  |  |
|  | rs2301052 | C/T | Controls | 4881(2259/2622) | 3733(76.5) | 1075(22.0) | 73(1.5) | 12.5 (11.9-13.2) | 1.08(0.95-1.24) | 0.24 |
|  |  |  | T2D | 3440(2041/1399) | 2593(75.4) | 783(22.8) | 64(1.9) | 13.2 (12.4-14.1) |  |  |
|  | rs16859825 | T/C | Controls | 4889(2268/2621) | 4184(85.6) | 680(13.9) | 25(0.5) | 7.5 (7.0-8.0) | 0.97(0.82-1.15) | 0.70 |
|  |  |  | T2D | 3510(2082/1428) | 3038(86.6) | 454(12.9) | 18(0.5) | 7.0 (6.4-7.6) |  |  |
|  | rs6783938 | C/T | Controls | 4901(2272/2629) | 4009(81.8) | 851(17.4) | 41(0.8) | 9.5 (8.9-10.1) | 1.09(0.94-1.27) | 0.26 |
|  |  |  | T2D | 3501(2079/1422) | 2850(81.4) | 616(17.6) | 35(1.0) | 9.8 (9.1-10.5) |  |  |
|  | rs6784193 | A/G | Controls | 4856(2246/2610) | 3456(71.2) | 1287(26.5) | 113(2.3) | 15.6 (14.9-16.3) | 1.14(1.01-1.29) | 0.03 |
|  |  |  | T2D | 3446(2060/1386) | 2396(69.5) | 953(27.7) | 97(2.8) | 16.6 (15.8-17.5) |  |  |
|  | rs6779662 | T/C | Controls | 4849(2256/2593) | 4219(87.0) | 604(12.5) | 26(0.5) | 6.8 (6.3-7.3) | 1.16(0.98-1.38) | 0.09 |
|  |  |  | T2D | 3430(2038/1392) | 2924(85.2) | 488(14.2) | 18(0.5) | 7.6 (7.0-8.3) |  |  |
|  | rs2160815 | T/A | Controls | 4890(2266/2624) | 3769(77.1) | 1046(21.4) | 75(1.5) | 12.2 (11.6-12.9) | 1.02(0.90-1.17) | 0.73 |
|  |  |  | T2D | 3453(2053/1400) | 2671(77.4) | 726(21.0) | 56(1.6) | 12.1 (11.4-12.9) |  |  |
|  | rs7635708 | A/G | Controls | 4877(2259/2618) | 4160(85.3) | 690(14.1) | 27(0.6) | 7.6 (7.1-8.2) | 1.20(1.02-1.41) | 0.03 |
|  |  |  | T2D | 3514(2082/1432) | 2940(83.7) | 546(15.5) | 28(0.8) | 8.6 (7.9-9.2) |  |  |
| *ECHS1* | rs7093778 | T/C | Controls | 4840(2249/2591) | 3159(65.3) | 1509(31.2) | 172(3.6) | 19.1 (18.4-19.9) | 0.98(0.88-1.10) | 0.79 |
|  |  |  | T2D | 3467(2059/1408) | 2297(66.3) | 1057(30.5) | 113(3.3) | 18.5 (17.6-19.4) |  |  |
|  | rs4838679 | T/C | Controls | 4813(2227/2586) | 2097(43.6) | 2128(44.2) | 588(12.2) | 34.3 (33.4-35.3) | 1.05(0.95-1.15) | 0.34 |
|  |  |  | T2D | 3473(2063/1410) | 1463(42.1) | 1592(45.8) | 418(12.0) | 35.0 (33.8-36.1) |  |  |
|  | rs11101721 | C/G | Controls | 4867(2252/2615) | 4451(91.5) | 407(8.4) | 9(0.2) | 4.4 (4.0-4.8) | 1.16(0.93-1.44) | 0.18 |
|  |  |  | T2D | 3513(2083/1430) | 3192(90.9) | 317(9.0) | 4(0.1) | 4.6 (4.1-5.1) |  |  |
| *HADHA/B* | rs4665848 | T/G | Controls | NA | NA | NA | NA | NA | NA | NA |
|  |  |  | T2D | NA | NA | NA | NA | NA |  |  |
|  | rs892447 | A/G | Controls | 4860(2247/2613) | 2486(51.2) | 1960(40.3) | 414(8.5) | 28.7 (27.8-29.6) | 0.94(0.86-1.04) | 0.25 |
|  |  |  | T2D | 3484(2065/1419) | 1785(51.2) | 1402(40.2) | 297(8.5) | 28.6 (27.6-29.7) |  |  |
|  | rs2289019 | C/G | Controls | 4845(2242/2603) | 3068(63.3) | 1571(32.4) | 206(4.3) | 20.5 (19.7-21.3) | 0.95(0.86-1.06) | 0.39 |
|  |  |  | T2D | 3499(2080/1419) | 2213(63.2) | 1132(32.4) | 154(4.4) | 20.6 (19.6-21.5) |  |  |
|  | rs10177371 | T/C | Controls | 4857(2252/2605) | 2644(54.4) | 1855(38.2) | 358(7.4) | 26.5 (25.6-27.4) | 0.98(0.89-1.08) | 0.65 |
|  |  |  | T2D | 3499(2083/1416) | 1890(54.0) | 1364(39.0) | 245(7.0) | 26.5 (25.5-27.5) |  |  |
| *ACADL* | rs1396828 | T/C | Controls | 4884(2265/2619) | 1405(28.8) | 2390(48.9) | 1089(22.3) | 46.8 (45.8-47.8) | 1.0(0.91-1.09) | 0.94 |
|  |  |  | T2D | 3501(2080/1421) | 1015(29.0) | 1717(49.0) | 769(22.0) | 46.5 (45.3-47.7) |  |  |
|  | rs2286963 | T/G | Controls | 4822(2239/2583) | 2040(42.3) | 2160(44.8) | 622(12.9) | 35.3 (34.3-36.3) | 1.01(0.92-1.10) | 0.88 |
|  |  |  | T2D | 3430(2036/1394) | 1453(42.4) | 1515(44.2) | 462(13.5) | 35.6 (34.4-36.7) |  |  |
|  | rs263680 | A/G | Controls | 4840(2243/2597) | 4115(85.0) | 691(14.3) | 34(0.7) | 7.8(7.3-8.4) | 1.03(0.88-1.21) | 0.71 |
|  |  |  | T2D | 3483(2071/1412) | 2944(84.5) | 511(14.7) | 28(0.8) | 8.1(7.5-8.8) |  |  |

Data are number of individuals, divided into genotype groups (% in each group), and frequencies of the minor allele (MAF) in percentages. Logistic regression was used to compare allele frequencies. The odds ratios (OR) and the 95% confidence interval (CI) are given for comparison of allele frequency. ORs and *P*-values shown are for an additive genetic model (*P_additive_)* and are adjusted for age, sex, and BMI. WT, wild-type. HE, heterozygous. HO, homozygous.

Genotype distribution and allele frequencies among lean controls and centrally obese individuals

| Gene | SNP | Major/  minor | Controls/  Obese | n (men/women) | WT | HE | HO | MAF (%) | OR (95% CI) | *P_additive_* |
| --- | --- | --- | --- | --- | --- | --- | --- | --- | --- | --- |
| *EHHADH* | rs2216386 | A/G | Controls | 4322(2386/1936) | 2725(63.0) | 1420(32.9) | 177(4.1) | 20.5 (19.7-21.4) | 0.98(0.90-1.05) | 0.51 |
|  |  |  | Obese | 6753(3304/3449) | 4304(63.7) | 2184(32.3) | 265(3.9) | 20.1 (19.4-20.8) |  |  |
|  | rs2301052 | C/T | Controls | 4338(2402/1936) | 3280(75.6) | 986(22.7) | 72(1.7) | 13.0 (12.3-13.8) | 0.98(0.89-1.07) | 0.62 |
|  |  |  | Obese | 6753(3312/3441) | 5133(76.0) | 1520(22.5) | 100(1.5) | 12.7 (12.2-13.3) |  |  |
|  | rs16859825 | T/C | Controls | 4356(2416/1940) | 3743(85.9) | 591(13.6) | 22(0.5) | 7.3 (6.8-7.9) | 0.96(0.85-1.07) | 0.45 |
|  |  |  | Obese | 6930(3416/3514) | 5988(86.4) | 913(13.2) | 29(0.4) | 7.0 (6.6-7.4) |  |  |
|  | rs6783938 | C/T | Controls | 4372(2424/1948) | 3555(81.3) | 776(17.7) | 41(0.9) | 9.8 (9.2-10.5) | 0.99(0.89-1.09) | 0.81 |
|  |  |  | Obese | 6936(3411/3525) | 5630(81.2) | 1249(18.0) | 57(0.8) | 9.8 (9.3-10.3) |  |  |
|  | rs6784193 | A/G | Controls | 4322(2387/1935) | 3024(70) | 1189(27.5) | 109(2.5) | 16.3 (15.5-17.1) | 0.97(0.90-1.06) | 0.51 |
|  |  |  | Obese | 6757(3327/3430) | 4765(70.5) | 1824(27) | 168(2.5) | 16.0 (15.4-16.6) |  |  |
|  | rs6779662 | T/C | Controls | 4291(2375/1916) | 3678(85.7) | 591(13.8) | 22(0.5) | 7.4 (6.9-8.0) | 0.93(0.83-1.05) | 0.26 |
|  |  |  | Obese | 6763(3326/3437) | 5840(86.4) | 894(13.2) | 29(0.4) | 7.0 (6.6-7.5) |  |  |
|  | rs2160815 | T/A | Controls | 4326(2387/1939) | 3363(77.7) | 890(20.6) | 73(1.7) | 12.0 (11.3-12.7) | 0.98(0.89-1.08) | 0.68 |
|  |  |  | Obese | 6761(3316/3445) | 5294(78.3) | 1380(20.4) | 87(1.3) | 11.5 (11.0-12.0) |  |  |
|  | rs7635708 | A/G | Controls | 4350(2410/1940) | 3674(84.5) | 653(15.0) | 23(0.5) | 8.0 (7.5-8.6) | 0.98(0.88-1.10) | 0.78 |
|  |  |  | Obese | 6940(3406/3534) | 5894(84.9) | 1001(14.4) | 45(0.6) | 7.9 (7.4-8.3) |  |  |
| *ECHS1* | rs7093778 | T/C | Controls | 4322(2404/1918) | 2854(66.0) | 1317(30.5) | 151(3.5) | 18.7 (17.9-19.6) | 1.03(0.95-1.11) | 0.51 |
|  |  |  | Obese | 6852(3366/3486) | 4484(65.4) | 2128(31.1) | 240(3.5) | 19.0 (18.4-19.7) |  |  |
|  | rs4838679 | T/C | Controls | 4316(2389/1927) | 1858(43.0) | 1910(44.3) | 548(12.7) | 34.8 (33.8-35.8) | 1.01(0.94-1.07) | 0.85 |
|  |  |  | Obese | 6877(3379/3498) | 2947(42.9) | 3114(45.3) | 816(11.9) | 34.5 (33.7-35.3) |  |  |
|  | rs11101721 | C/G | Controls | 4337(2400/1937) | 3973(91.6) | 357(8.2) | 7(0.2) | 4.3 (3.9-4.7) | 1.21(1.05-1.40) | 0.008 |
|  |  |  | Obese | 6975(3430/3545) | 6294(90.2) | 666(9.5) | 15(0.2) | 5.0 (4.6-5.4) |  |  |
| *HADHA/B* | rs4665848 | T/G | Controls | NA | NA | NA | NA | NA | NA | NA |
|  |  |  | Obese | NA | NA | NA | NA | NA |  |  |
|  | rs892447 | A/G | Controls | 4348(2405/1943) | 2201(50.6) | 1759(40.5) | 388(8.9) | 29.2 (28.2-30.1) | 0.93(0.87-0.99) | 0.03 |
|  |  |  | Obese | 6897(3377/3520) | 3574(51.8) | 2790(40.5) | 533(7.7) | 28.0 (27.2-28.7) |  |  |
|  | rs2289019 | C/G | Controls | 4348(2408/1940) | 2756(63.4) | 1405(32.3) | 187(4.3) | 20.5 (19.6-21.3) | 1.0(0.93-1.07) | 0.93 |
|  |  |  | Obese | 6926(3405/3521) | 4396(63.5) | 2228(32.2) | 302(4.4) | 20.4 (19.8-21.1) |  |  |
|  | rs10177371 | T/C | Controls | 4332(2409/1923) | 2376(54.8) | 1645(38.0) | 311(7.2) | 26.2 (25.2-27.1) | 1.0(0.94-1.07) | 0.97 |
|  |  |  | Obese | 6890(3391/3499) | 3752(54.5) | 2673(38.8) | 465(6.7) | 26.1 (25.4-26.9) |  |  |
| *ACADL* | rs1396828 | T/C | Controls | 4350(2414/1936) | 1236(28.4) | 2154(49.5) | 960(22.1) | 46.8 (45.8-47.9) | 0.98(0.92-1.04) | 0.45 |
|  |  |  | Obese | 6923(3401/3522) | 2040(29.5) | 3390(49.0) | 1493(21.6) | 46.0 (45.2-46.9) |  |  |
|  | rs2286963 | T/G | Controls | 4277(2367/1910) | 1805(42.2) | 1921(44.9) | 551(12.9) | 35.3 (34.3-36.4) | 1.05(0.99-1.12) | 0.13 |
|  |  |  | Obese | 6726(3301/3425) | 2787(41.4) | 3001(44.6) | 938(13.9) | 36.3 (35.4-37.1) |  |  |
|  | rs263680 | A/G | Controls | 4333(2401/1932) | 3678(84.9) | 624(14.4) | 31(0.7) | 7.9(7.4-8.5) | 0.99(0.89-1.1) | 0.86 |
|  |  |  | Obese | 6883(3379/3504) | 5821(84.6) | 1009(14.7) | 53(0.8) | 8.1(7.6-8.6) |  |  |

Data are number of individuals, divided into genotype groups (% in each group), and frequencies of the minor allele (MAF) in percentages. Logistic regression was used to compare allele frequencies. The odds ratios (OR) and the 95% confidence interval (CI) are given for comparison of allele frequency. ORs and *P*-values shown are for an additive genetic model (*P_additive_)* and are adjusted for age, sex, and diabetes treatment. WT, wild-type. HE, heterozygous. HO, homozygous.

**Genotype distribution and allele frequencies among control individuals with no components of MetS and individuals with MetS**

| Gene | SNP | Major/  minor | Controls/  Obese | n (men/women) | WT | HE | HO | MAF (%) | OR (95% CI) | *P_additive_* |
| --- | --- | --- | --- | --- | --- | --- | --- | --- | --- | --- |
| *EHHADH* | rs2216386 | A/G | Controls | 1695(489/1206) | 1088(64.2) | 545(32.2) | 62(3.7) | 19.7 (18.4-21.1) | 1.11(0.96-1.29) | 0.14 |
|  |  |  | MetS | 1319(891/428) | 844(64.0) | 418(31.7) | 57(4.3) | 20.2 (18.7-21.7) |  |  |
|  | rs2301052 | C/T | Controls | 1700(491/1209) | 1296(76.2) | 384(22.6) | 20(1.2) | 12.5 (11.4-13.6) | 1.07(0.89-1.27) | 0.47 |
|  |  |  | MetS | 1310(884/426) | 1017(77.6) | 265(20.2) | 28(2.1) | 12.3 (11.0-13.6) |  |  |
|  | rs16859825 | T/C | Controls | 1701(491/1210) | 1473(86.6) | 219(12.9) | 9(0.5) | 7.0 (6.1-7.9) | 1.14(0.91-1.42) | 0.26 |
|  |  |  | MetS | 1319(891/428) | 1129(85.6) | 180(13.6) | 10(0.8) | 7.6 (6.6-8.7) |  |  |
|  | rs6783938 | C/T | Controls | 1703(494/1209) | 1414(83.0) | 276(16.2) | 13(0.8) | 8.9 (7.9-9.9) | 1.25(1.03-1.52) | 0.02 |
|  |  |  | MetS | 1323(893/430) | 1068(80.7) | 239(18.1) | 16(1.2) | 10.2 (9.1-11.5) |  |  |
|  | rs6784193 | A/G | Controls | 1691(494/1197) | 1207(71.4) | 450(26.6) | 34(2.0) | 15.3 (14.1-16.6) | 1.13(0.97-1.33) | 0.12 |
|  |  |  | MetS | 1304(881/423) | 927(71.1) | 336(25.8) | 41(3.1) | 16.0 (14.6-17.5) |  |  |
|  | rs6779662 | T/C | Controls | 1687(494/1193) | 1464(86.8) | 214(12.7) | 9(0.5) | 6.9 (6.0-7.8) | 1.14(0.91-1.43) | 0.25 |
|  |  |  | MetS | 1299(876/423) | 1126(86.7) | 161(12.4) | 12(0.9) | 7.1 (6.2-8.2) |  |  |
|  | rs2160815 | T/A | Controls | 1703(497/1206) | 1332(78.2) | 339(19.9) | 32(1.9) | 11.8 (10.8-13.0) | 1.02(0.85-1.22) | 0.82 |
|  |  |  | MetS | 1320(891/429) | 1029(78.0) | 272(20.6) | 19(1.4) | 11.7 (10.5-13.0) |  |  |
|  | rs7635708 | A/G | Controls | 1694(493/1201) | 1438(84.9) | 247(14.6) | 9(0.5) | 7.8 (6.9-8.8) | 1.08(0.87-1.34) | 0.49 |
|  |  |  | MetS | 1320(889/431) | 1125(85.2) | 183(13.9) | 12(0.9) | 7.8 (6.8-8.9) |  |  |
| *ECHS1* | rs7093778 | T/C | Controls | 1680(491/1189) | 1102(65.6) | 520(31.0) | 58(3.5) | 18.9 (17.6-20.3) | 1.08(0.94-1.25) | 0.28 |
|  |  |  | MetS | 1308(885/423) | 856(65.4) | 388(29.7) | 64(4.9) | 19.7 (18.2-21.3) |  |  |
|  | rs4838679 | T/C | Controls | 1691(490/1201) | 737(43.6) | 757(44.8) | 197(11.6) | 34.0 (32.4-35.7) | 1.05(0.93-1.18) | 0.44 |
|  |  |  | MetS | 1309(883/426) | 556(42.5) | 583(44.5) | 170(13.0) | 35.3 (33.4-37.1) |  |  |
|  | rs11101721 | C/G | Controls | 1695(492/1203) | 1558(91.9) | 133(7.8) | 4(0.2) | 4.2 (3.5-4.9) | 1.35(1.03-1.78) | 0.03 |
|  |  |  | MetS | 1315(889/426) | 1187(90.3) | 122(9.3) | 6(0.5) | 5.1 (4.3-6.0) |  |  |
| *HADHA/B* | rs4665848 | T/G | Controls | NA | NA | NA | NA | NA | NA | NA |
|  |  |  | MetS | NA | NA | NA | NA | NA |  |  |
|  | rs892447 | A/G | Controls | 1696(495/1201) | 882(52.0) | 679(40.0) | 135(8.0) | 28.0 (26.5-29.5) | 0.95(0.84-1.08) | 0.45 |
|  |  |  | MetS | 1306(879/427) | 695(53.2) | 505(38.7) | 106(8.1) | 27.5 (25.7-29.2) |  |  |
|  | rs2289019 | C/G | Controls | 1690(489/1201) | 1072(63.4) | 555(32.8) | 63(3.7) | 20.1 (18.8-21.5) | 0.94(0.82-1.09) | 0.45 |
|  |  |  | MetS | 1315(890/425) | 851(64.7) | 408(31.0) | 56(4.3) | 19.8 (18.3-21.3) |  |  |
|  | rs10177371 | T/C | Controls | 1688(491/1197) | 938(55.6) | 636(37.7) | 114(6.8) | 25.6 (24.1-27.1) | 0.99(0.87-1.13) | 0.90 |
|  |  |  | MetS | 1311(885/426) | 721(55.0) | 497(37.9) | 93(7.1) | 26.0 (24.4-27.8) |  |  |
| *ACADL* | rs1396828 | T/C | Controls | 1698(496/1202) | 499(29.4) | 808(47.6) | 391(23.0) | 46.8 (45.1-48.5) | 0.94(0.84-1.06) | 0.33 |
|  |  |  | MetS | 1311(883/428) | 361(27.5) | 670(51.1) | 280(21.4) | 46.9 (45.0-48.8) |  |  |
|  | rs2286963 | T/G | Controls | 1679(490/1189) | 708(42.2) | 746(44.4) | 225(13.4) | 35.6 (34.0-37.3) | 1.09(0.97-1.23) | 0.15 |
|  |  |  | MetS | 1301(882/419) | 524(40.3) | 602(46.3) | 175(13.5) | 36.6 (34.7-38.5) |  |  |
|  | rs263680 | A/G | Controls | 4500(1919/2581) | 3835(85.2) | 635(14.1) | 30(0.7) | 7.7(7.2-8.3) | 1.04(0.90-1.19) | 0.60 |
|  |  |  | MetS | 2546(1668/878) | 2163(85.0) | 365(14.3) | 18(0.7) | 7.9(7.1-8.6) |  |  |

Data are number of individuals, divided into genotype groups (% in each group), and frequencies of the minor allele (MAF) in percentages. Logistic regression was used to compare allele frequencies. The odds ratios (OR) and the 95% confidence interval (CI) are given for comparison of allele frequency. ORs and *P*-values shown are for an additive genetic model (*P_additive_)* and are adjusted for age and sex. WT, wild-type. HE, heterozygous. HO, homozygous.

Quantitative metabolic traits among glucose-tolerant individuals in the population-based Inter99 study sample in relation to *EHHADH* genotypes

| ***EHHADH:* rs2216386** |  |  |  |  |  |
| --- | --- | --- | --- | --- | --- |
|  | A/A | A/G | G/G | Per allele effect (95% CI) | *P* _additive_ |
| *n* (men/women) | 2777 (1294/1483) | 1406 (640/766) | 180 (84/96) |  |  |
| Age (years) | 45±8 | 45±8 | 44±8 |  |  |
| BMI (kg/m^2^) | 25.5±4.1 | 25.4±4.0 | 25.8±4.5 | 0.05 (-0.16; 0.27) | 0.61 |
| Waist circumference (cm) | 84.2±12.2 | 83.9±12.2 | 85.0±12.8 | -0.04 (-0.31; 0.23) | 0.77 |
| Triglyceride (mmol/l) | 1.0 (0.7-1.4) | 1.0 (0.7-1.4) | 1.0 (0.7-1.5) | 1.0% (-2.0%; 3.8%) | 0.43 |
| Fasting plasma glucose (mmol/l) | 5.3±0.4 | 5.3±0.4 | 5.3±0.4 | 0.001 (-0.018; 0.021) | 0.90 |
| Fasting serum insulin (pmol/l) | 31 (22-44) | 32 (23-46) | 36 (25-53) | 3.4% (0.8%; 6.1%) | 0.01 |
| ***EHHADH:* rs2301052** |  |  |  |  |  |
|  | C/C | C/T | T/T | Per allele effect (95% CI) | *P* _additive_ |
| *n* (men/women) | 3352 (1557/1795) | 962 (436/526) | 64 (30/34) |  |  |
| Age (years) | 45±8 | 45±8 | 43±7 |  |  |
| BMI (kg/m^2^) | 25.5±4.1 | 25.4±4.0 | 26.0±4.3 | -0.02 (-0.28; 0.23) | 0.86 |
| Waist circumference (cm) | 84.3±12.1 | 83.5±12.4 | 85.3±12.4 | -0.28 (-0.61; 0.05) | 0.09 |
| Triglyceride (mmol/l) | 1.0 (0.7-1.4) | 1.0 (0.7-1.3) | 1.0 (0.8-1.8) | 0.9% (-2.0%; 3.8%) | 0.53 |
| Fasting plasma glucose (mmol/l) | 5.3±0.4 | 5.3±0.4 | 5.3±0.4 | -0.02 (-0.04; 0.00) | 0.11 |
| Fasting serum insulin (pmol/l) | 31 (22-45) | 31 (22-46) | 37 (22-56) | 0.8% (-2.4%; 4.0%) | 0.62 |
| ***EHHADH:* rs16859825** |  |  |  |  |  |
|  | T/T | T/C | C/C | Per allele effect (95% CI) | *P* _additive_ |
| *n* (men/women) | 3747 (1731/2016) | 613 (295/318) | 24 (7/17) |  |  |
| Age (years) | 45±8 | 45±8 | 45±9 |  |  |
| BMI (kg/m^2^) | 25.5±4.0 | 25.6±4.2 | 25.9±3.6 | 0.13 (-0.19; 0.45) | 0.43 |
| Waist circumference (cm) | 84.0±12.2 | 84.8±12.1 | 84.1±11.0 | 0.37 (-0.03; 0.78) | 0.07 |
| Triglyceride (mmol/l) | 1.0 (0.7-1.4) | 1.0 (0.7-1.4) | 1.0 (0.6-1.2) | 0.9% (-2.7%; 4.5%) | 0.63 |
| Fasting plasma glucose (mmol/l) | 5.3±0.4 | 5.3±0.4 | 5.3±0.4 | 0.033 (0.004; 0.062) | 0.03 |
| Fasting serum insulin (pmol/l) | 31 (22-45) | 34 (24-48) | 31 (26-49) | 5.3% (1.4%; 9.3%) | 0.01 |
| ***EHHADH:* rs6783938** |  |  |  |  |  |
|  | C/C | C/T | T/T | Per allele effect (95% CI) | *P* _additive_ |
| *n* (men/women) | 3596 (1655/1941) | 765 (363/402) | 39 (19/20) |  |  |
| Age (years) | 45±8 | 45±8 | 45±8 |  |  |
| BMI (kg/m^2^) | 25.5±4.1 | 25.4±4.1 | 26.8±5.9 | 0.06 (-0.23; 0.34) | 0.70 |
| Waist circumference (cm) | 84.2±12.2 | 84.3±12.3 | 86.8±13.1 | 0.09 (-0.28; 0.45) | 0.65 |
| Triglyceride (mmol/l) | 1.0 (0.7-1.4) | 1.0 (0.7-1.4) | 1.0 (0.6-1.3) | 0.6% (-2.6%; 3.8%) | 0.71 |
| Fasting plasma glucose (mmol/l) | 5.3±0.4 | 5.3±0.4 | 5.3±0.3 | 0.009 (-0.018; 0.035) | 0.52 |
| Fasting serum insulin (pmol/l) | 31 (22-45) | 34 (23-48) | 31.00 (26-48) | 4.1% (0.5%; 7.7%) | 0.03 |
| ***EHHADH:* rs6784193** |  |  |  |  |  |
|  | A/A | A/G | G/G | Per allele effect (95% CI) | *P* _additive_ |
| *n* (men/women) | 3089 (1435/1654) | 1157 (521/636) | 107 (55/52) |  |  |
| Age (years) | 45±8 | 45±8 | 44±7 |  |  |
| BMI (kg/m^2^) | 25.5±4.1 | 25.3±4.0 | 26.2±4.8 | -0.03 (-0.27; 0.20) | 0.77 |
| Waist circumference (cm) | 84.3±12.1 | 83.6±12.2 | 85.9±13.2 | -0.10 (-0.40; 0.20) | 0.50 |
| Triglyceride (mmol/l) | 1.0 (0.7-1.4) | 1.0 (0.7-1.4) | 1.0 (0.7-1.6) | 1.7% (-0.9%; 4.3%) | 0.19 |
| Fasting plasma glucose (mmol/l) | 5.3±0.4 | 5.3±0.4 | 5.4±0.4 | 0.01 (-0.02; 0.023) | 0.64 |
| Fasting serum insulin (pmol/l) | 31 (22-45) | 33 (23-47) | 33 (26-56) | 3.8% (0.8%; 6.7%) | 0.01 |
| ***EHHADH:* rs6779662** |  |  |  |  |  |
|  | T/T | T/C | C/C | Per allele effect (95% CI) | *P* _additive_ |
| *n* (men/women) | 3786 (1763/2023) | 542 (249/293) | 21 (10/11) |  |  |
| Age (years) | 45±8 | 45±8 | 46±7 |  |  |
| BMI (kg/m^2^) | 25.5±4.1 | 25.2±4.0 | 26.9±4.9 | -0.16 (-0.50; 0.18) | 0.36 |
| Waist circumference (cm) | 84.4±12.2 | 82.9±12.3 | 87.1±13.0 | -0.60 (-1.03; -0.17) | 0.006 |
| Triglyceride (mmol/l) | 1.0 (0.7-1.4) | 1.0 (0.8-1.4) | 0.9 (0.6-1.8) | 2.1% (-1.7%; 5.9%) | 0.28 |
| Fasting plasma glucose (mmol/l) | 5.3±0.4 | 5.3±0.4 | 5.3±0.3 | -0.01 (-0.04; 0.02) | 0.42 |
| Fasting serum insulin (pmol/l) | 31 (22-45) | 31 (23-47) | 30 (22-56) | 0.03% (-0.02%; 0.07%) | 0.24 |
| ***EHHADH:* rs2160815** |  |  |  |  |  |
|  | T/T | T/A | A/A | Per allele effect (95% CI) | *P* _additive_ |
| *n* (men/women) | 3397 (1563/1834) | 925 (442/483) | 67 (28/39) |  |  |
| Age (years) | 45±8 | 45±8 | 44±7 |  |  |
| BMI (kg/m^2^) | 25.5±4.1 | 25.7±4.1 | 24.7±3.7 | 0.07 (-0.19; 0.33) | 0.59 |
| Waist circumference (cm) | 84.2±12.2 | 84.6±12.1 | 81.5±11.8 | -0.17 (-0.50; 0.15) | 0.30 |
| Triglyceride (mmol/l) | 1.0 (0.7-1.4) | 1.0 (0.8-1.5) | 1.0 (0.7-1.2) | 3.2% (0.3%; 6.1%) | 0.03 |
| Fasting plasma glucose (mmol/l) | 5.3±0.4 | 5.3±0.4 | 5.2±0.4 | -0.02 (-0.04; 0.01) | 0.13 |
| Fasting serum insulin (pmol/l) | 31 (22-45) | 32 (23-47) | 30 (20-40) | -0.05% (-3.28; 3.19%) | 0.98 |
| ***EHHADH:* rs7635708** |  |  |  |  |  |
|  | A/A | A/G | G/G | Per allele effect (95% CI) | *P* _additive_ |
| *n* (men/women) | 3719 (1735/1984) | 627 (271/356) | 26 (17/9) |  |  |
| Age (years) | 45±8 | 45±8 | 45±6 |  |  |
| BMI (kg/m^2^) | 25.5±4.1 | 25.3±4.0 | 27.1±4.7 | -0.00 (-0.32; 0.31) | 0.98 |
| Waist circumference (cm) | 84.3±12.1 | 83.2±12.6 | 89.1±13.1 | -0.25 (-0.65; 0.15) | 0.21 |
| Triglyceride (mmol/l) | 1.0 (0.7-1.4) | 1.0 (0.7-1.4) | 0.8 (0.7-1.8) | 2.1% (-1.5%; 5.6%) | 0.26 |
| Fasting plasma glucose (mmol/l) | 5.3±0.4 | 5.3±0.4 | 5.5±0.4 | -0.005 (-0.034; 0.024) | 0.72 |
| Fasting serum insulin (pmol/l) | 31 (22-45) | 31 (23-47) | 36 (22-57) | 2.0% (-1.9%; 6.0%) | 0.32 |

Unadjusted mean±S.D. or medians (interquartile range) for a total of 4,567 middle-aged glucose-tolerant individuals stratified according to genotype. Values of serum triglycerides and serum insulin were logarithmically transformed prior to statistical analyses, and their effect sizes are presented as the increase/decrease in percent. Effect sizes and *P*-values shown are for an additive genetic model and are adjusted for age, sex, and BMI.

Quantitative metabolic traits among glucose-tolerant individuals in the population-based Inter99 study sample in relation to *ECHS1* genotypes

| ***ECHS1:* rs7093778** |  |  |  |  |  |
| --- | --- | --- | --- | --- | --- |
|  | T/T | T/C | C/C | Per allele effect (95% CI) | *P* _additive_ |
| *n* (men/women) | 840 (1335/1505) | 1350 (612/738) | 149 (67/82) |  |  |
| Age (years) | 45±8 | 45±8 | 45±7 |  |  |
| BMI (kg/m^2^) | 25.5±4.2 | 25.5±3.9 | 25.5±3.8 | 0.05 (-0.17; 0.26) | 0.67 |
| Waist circumference (cm) | 84.2±12.4 | 84.2±11.8 | 84.0±11.5 | 0.07 (-0.20; 0.35) | 0.61 |
| Triglyceride (mmol/l) | 1.0 (0.7-1.4) | 1.0 (0.7-1.4) | 1.0 (0.7-1.3) | 1.0% (-1.4%; 3.5%) | 0.40 |
| Fasting plasma glucose (mmol/l) | 5.3±0.4 | 5.3±0.4 | 5.4±0.4 | 0.03 (0.01; 0.05) | 0.002 |
| Fasting serum insulin (pmol/l) | 31 (22-45) | 32 (23-46) | 32 (23-44) | 0.9% (-1.8%; 3.7%) | 0.49 |
| ***ECHS1:* rs4838679** |  |  |  |  |  |
|  | T/T | T/C | C/C | Per allele effect (95% CI) | *P* _additive_ |
| *n* (men/women) | 1889 (887/1002) | 1916 (847/1069) | 525 (267/258) |  |  |
| Age (years) | 45±8 | 45±8 | 45±8 |  |  |
| BMI (kg/m^2^) | 25.5±4.0 | 25.5±4.2 | 25.2±4.2 | -0.11 (-0.28; 0.07) | 0.24 |
| Waist circumference (cm) | 84.4±12.2 | 84.0±12.3 | 83.8±11.8 | -0.07 (-0.30; 0.15) | 0.52 |
| Triglyceride (mmol/l) | 1.0 (0.7-1.4) | 1.0 (0.7-1.4) | 1.0 (0.7-1.4) | 0.9% (-1.1%; 2.9%) | 0.36 |
| Fasting plasma glucose (mmol/l) | 5.3±0.4 | 5.3±0.4 | 5.3±0.4 | -0.00 (-0.02; 0.02) | 0.99 |
| Fasting serum insulin (pmol/l) | 31 (22-45) | 31 (23-45) | 31 (22-46) | 0.6% (-1.7%; 2.8%) | 0.63 |
| ***ECHS1:* rs11101721** |  |  |  |  |  |
|  | C/C | C/G | G/G | Per allele effect (95% CI) | *P* _additive_ |
| *n* (men/women) | 3984 (1842/2142) | 370 (174/196) | 9 (2/7) |  |  |
| Age (years) | 45±8 | 45±8 | 44±6 |  |  |
| BMI (kg/m^2^) | 25.5±4.1 | 25.4±4.1 | 25.5±2.4 | -0.06 (-0.47; 0.35) | 0.77 |
| Waist circumference (cm) | 84.2±12.2 | 84.1±12.1 | 83.1±8.5 | 0.14 (-0.38; 0.66) | 0.59 |
| Triglyceride (mmol/l) | 1.0 (0.7-1.4) | 1.0 (0.7-1.4) | 0.8 (0.6-1.5) | 1.5% (-3.0%; 6.1%) | 0.51 |
| Fasting plasma glucose (mmol/l) | 5.3±0.4 | 5.3±0.4 | 5.2±0.5 | 0.02 (-0.02; 0.06) | 0.36 |
| Fasting serum insulin (pmol/l) | 31 (22-46) | 31 (22-46) | 40 (24-58) | -0.3% (-5.4; 4.8%) | 0.90 |

Unadjusted mean±S.D. or medians (interquartile range) for a total of 4,567 middle-aged glucose-tolerant individuals stratified according to genotype. Values of serum triglycerides and serum insulin were logarithmically transformed prior to statistical analyses, and their effect sizes are presented as the increase/decrease in percent. Effect sizes and *P*-values shown are for an additive genetic model and are adjusted for age, sex, and BMI.

Quantitative metabolic traits among glucose-tolerant individuals in the population-based Inter99 study sample in relation to *HADHA/B* genotypes

| ***HADHA/B:* rs4665848** |  |  |  |  |  |
| --- | --- | --- | --- | --- | --- |
|  | T/T | T/G | G/G | Per allele effect (95% CI) | *P*_dominant_ |
| *n* (men/women) | 4340 (2006/2334) | 73 (38/35) |  |  |  |
| Age (years) | 45±8 | 44±8 |  |  |  |
| BMI (kg/m^2^) | 25.5±4.1 | 25.9±4.5 |  | 0.37 (-0.57; 1.30) | 0.44 |
| Waist circumference (cm) | 84.1±12.2 | 85.7±11.8 |  | 0.19 (-0.99; 1.37) | 0.75 |
| Triglyceride (mmol/l) | 1.0 (0.7-1.4) | 1.0 (0.8-1.4) |  | -0.6% (-11.1%; 9.8%) | 0.91 |
| Fasting plasma glucose (mmol/l) | 5.3±0.4 | 5.3±0.4 |  | -0.05 (-0.14; 0.03) | 0.22 |
| Fasting serum insulin (pmol/l) | 31 (22-45) | 37 (25-52) |  | 5.8% (-5.8%; 17.4%) | 0.32 |
| ***HADHA/B:* rs892447** |  |  |  |  |  |
|  | A/A | A/G | G/G | Per allele effect (95% CI) | *P* _additive_ |
| *n* (men/women) | 2242 (1040/1202) | 1760 (792/968) | 355 (180/175) |  |  |
| Age (years) | 45±8 | 45±8 | 46±8 |  |  |
| BMI (kg/m^2^) | 25.5±4.0 | 25.5±4.2 | 25.5±4.2 | -0.009 (-0.197; 0.178) | 0.92 |
| Waist circumference (cm) | 84.3±12.1 | 83.9±12.3 | 84.7±12.2 | -0.20 (-0.44; 0.03) | 0.09 |
| Triglyceride (mmol/l) | 1.0 (0.7-1.4) | 1.0 (0.7-1.4) | 1.0 (0.7-1.4) | 0.9% (-1.2%; 3.0%) | 0.38 |
| Fasting plasma glucose (mmol/l) | 5.3±0.4 | 5.3±0.4 | 5.3±0.4 | 0.01 (-0.01; 0.03) | 0.23 |
| Fasting serum insulin (pmol/l) | 32 (23-46) | 31 (22-45) | 31 (23-44) | -1.7% (-4.0%; 0.7%) | 0.16 |
| ***HADHA/B:* rs2289019** |  |  |  |  |  |
|  | C/C | C/G | G/G | Per allele effect (95% CI) | *P* _additive_ |
| *n* (men/women) | 2767 (1292/1475) | 1413 (634/779) | 168 (85/83) |  |  |
| Age (years) | 45±8 | 45±8 | 46±8 |  |  |
| BMI (kg/m^2^) | 25.5±4.0 | 25.4±4.1 | 26.0±4.8 | 0.06 (-0.16; 0.27) | 0.60 |
| Waist circumference (cm) | 84.2±12.1 | 83.8±12.2 | 85.8±13.0 | -0.10 (-0.37; 0.17) | 0.46 |
| Triglyceride (mmol/l) | 1.0 (0.7-1.4) | 1.0 (0.7-1.4) | 1.0 (0.7-1.4) | 0.8% (-1.6%; 3.1%) | 0.54 |
| Fasting plasma glucose (mmol/l) | 5.3±0.4 | 5.3±0.4 | 5.3±0.4 | 0.01 (-0.01; 0.03) | 0.41 |
| Fasting serum insulin (pmol/l) | 32 (23-46) | 30 (22-45) | 32 (26-47) | -1.3% (-3.9%; 1.4%) | 0.36 |
| ***HADHA/B:* rs10177371** |  |  |  |  |  |
|  | T/T | T/C | C/C | Per allele effect (95% CI) | *P* _additive_ |
| *n* (men/women) | 2382 (1103/1279) | 1664 (755/909) | 309 (160/149) |  |  |
| Age (years) | 45±8 | 45±8 | 46±8 |  |  |
| BMI (kg/m^2^) | 25.4±3.9 | 25.6±4.1 | 25.7±4.5 | 0.12 (-0.07; 0.31) | 0.23 |
| Waist circumference (cm) | 84.1±12.1 | 84.2±12.3 | 84.8±12.3 | -0.20 (-0.45; 0.04) | 0.10 |
| Triglyceride (mmol/l) | 1.0 (0.7-1.4) | 1.0 (0.7-1.4) | 1.0 (0.7-1.4) | 1.1% (-1.0%; 3.3%) | 0.30 |
| Fasting plasma glucose (mmol/l) | 5.3±0.4 | 5.3±0.4 | 5.3±0.4 | 0.002 (-0.016; 0.019) | 0.84 |
| Fasting serum insulin (pmol/l) | 31 (22-45) | 31 (22-46) | 32 (25-48) | 0.04% (-0.31%; 0.38%) | 0.46 |

Unadjusted mean±S.D. or medians (interquartile range) for a total of 4,567 middle-aged glucose-tolerant individuals stratified according to genotype. Values of serum triglycerides and serum insulin were logarithmically transformed prior to statistical analyses, and their effect sizes are presented as the increase/decrease in percent. Effect sizes and *P*-values shown are for an additive genetic model and are adjusted for age, sex, and BMI.

Quantitative metabolic traits among glucose-tolerant individuals in the population-based Inter99 study sample in relation to *ACADL* genotypes

| ***ACADL:* rs1396828** |  |  |  |  |  |
| --- | --- | --- | --- | --- | --- |
|  | T/T | T/C | C/C | Per allele effect (95% CI) | *P* _additive_ |
| *n* (men/women) | 1267 (546/721) | 2139 (997/1142) | 974 (486/488) |  |  |
| Age (years) | 45±8 | 45±8 | 45±8 |  |  |
| BMI (kg/m^2^) | 25.4±4.0 | 25.6±4.0 | 25.5±4.2 | 0.04 (-0.13; 0.21) | 0.66 |
| Waist circumference (cm) | 83.9±12.5 | 84.3±11.9 | 84.3±12.2 | -0.3 (-0.5; -0.1) | 0.01 |
| Triglyceride (mmol/l) | 1.0 (0.7-1.4) | 1.0 (0.7-1.4) | 1.0 (0.7-1.4) | -1.5% (-3.4%; 0.4%) | 0.12 |
| Fasting plasma glucose (mmol/l) | 5.3±0.4 | 5.3±0.4 | 5.3±0.4 | -0.00 (-0.02; 0.01) | 0.79 |
| Fasting serum insulin (pmol/l) | 31 (22-45) | 31 (23-46) | 31 (22-45) | -0.1% (-2.2%; 2.0%) | 0.94 |
| ***ACADL:* rs2286963** |  |  |  |  |  |
|  | T/T | T/G | G/G | Per allele effect (95% CI) | *P* _additive_ |
| *n* (men/women) | 1825 (858/967) | 1933 (900/1033) | 569 (251/318) |  |  |
| Age (years) | 45±8 | 45±8 | 45±8 |  |  |
| BMI (kg/m^2^) | 25.5±4.2 | 25.5±3.9 | 25.5±4.2 | -0.01 (-0.19; 0.17) | 0.91 |
| Waist circumference (cm) | 84.1±12.3 | 84.3 ±11.8 | 84.3±12.8 | 0.26 (0.04; 0.48) | 0.02 |
| Triglyceride (mmol/l) | 1.0 (0.7-1.4) | 1.0 (0.8-1.4) | 1.0 (0.7-1.4) | 1.8% (-0.1%; 3.9%) | 0.06 |
| Fasting plasma glucose (mmol/l) | 5.3±0.4 | 5.3±0.4 | 5.3±0.4 | 0.002(-0.014; 0.018) | 0.78 |
| Fasting serum insulin (pmol/l) | 32 (23-46) | 31 (22-45) | 31 (22-46) | -1.0% (-3.1%; 1.2%) | 0.39 |
| ***ACADL:* rs263680** |  |  |  |  |  |
|  | A/A | A/G | G/G | Per allele effect (95% CI) | *P* _additive_ |
| *n* (men/women) | 3693 (1726/1967) | 627 (276/351) | 29 (14/15) |  |  |
| Age (years) | 45±8 | 45±8 | 47±8 |  |  |
| BMI (kg/m^2^) | 25.5±4.1 | 25.4±3.9 | 25.9±4.3 | -0.08 (-0.39; 0.23) | 0.62 |
| Waist circumference (cm) | 84.2±12.1 | 83.9 ±12.6 | 85.7±12.3 | 0.29 (-0.11; 0.69) | 0.16 |
| Triglyceride (mmol/l) | 1.1 (0.8-1.6) | 1.1 (0.8-1.6) | 1.2 (0.7-1.5) | 1.4% (-2.1%; 5.0%) | 0.43 |
| Fasting plasma glucose (mmol/l) | 5.3±0.4 | 5.3±0.4 | 5.3±0.4 | 0.001(-0.005; 0.006) | 0.82 |
| Fasting serum insulin (pmol/l) | 34 (24-52) | 36 (24-52) | 39 (27-56) | 1.9% (-2.0%; 5.8%) | 0.62 |

Unadjusted mean±S.D. or medians (interquartile range) for a total of 4,567 middle-aged glucose-tolerant individuals stratified according to genotype. Values of serum triglycerides and serum insulin were logarithmically transformed prior to statistical analyses, and their effect sizes are presented as the increase/decrease in percent. Effect sizes and *P*-values shown are for an additive genetic model and are adjusted for age, sex, and BMI.

**QQ-plot of observed versus expected *P*-values**

**
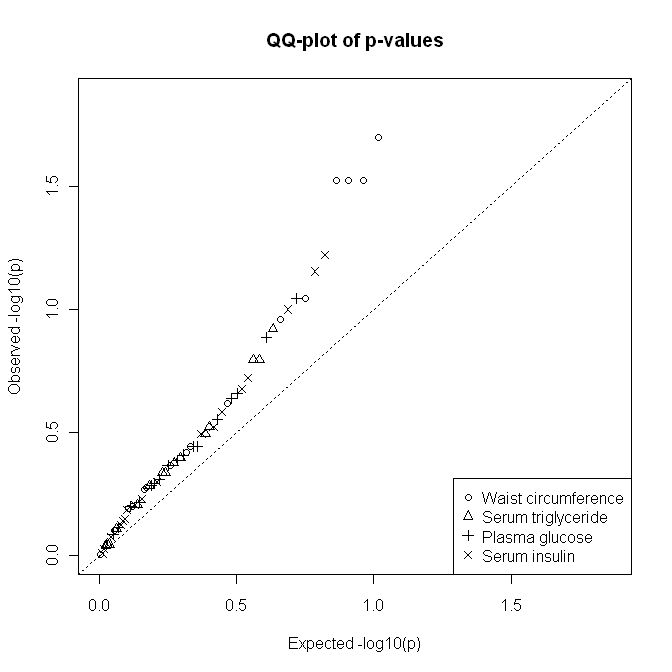
**

The plot shows observed (x-axis) and expected (y-axis) values of -log10 (*P*-values) resulting from the test for association between the 18 tagSNPs and waist circumference, serum triglyceride, plasma glucose, and serum insulin. The dotted line has a slope of 1 and an intercept of 0.

1. Without hyphens (””) [↑](#footnote-ref-2)
